# Global land footprint of UK food and feed imports under future socioeconomic scenarios

**DOI:** 10.1371/journal.pone.0352499

**Published:** 2026-06-30

**Authors:** Bartlomiej Arendarczyk, Sam Rabin, Daniel Bampoh, Almut Arneth, Calum Brown, Mark Rounsevell, Peter Alexander

**Affiliations:** 1 School of GeoSciences, Drummond Street, University of Edinburgh, Edinburgh, United Kingdom; 2 Climate & Global Dynamics Laboratory, National Center for Atmospheric Research, Boulder, Colorado, United States of America; 3 Institute of Meteorology and Climate Research, Atmospheric Environmental Research (IMK-IFU), Karlsruhe Institute of Technology, Kreuzeckbahnstraße, Garmisch-Partenkirchen, Germany; 4 Institute of Geography & Geo-ecology, Karlsruhe Institute of Technolog, Karlsruhe, Germany; 5 Global Academy of Agriculture and Food Systems, University of Edinburgh, Edinburgh, United Kingdom; Sichuan University, CHINA

## Abstract

Globalisation in the food system has led to interdependencies between countries for food security and has distributed the environmental impacts of the food consumption. In the UK, food imports account for nearly half of domestic consumption. However, there has been limited research quantifying the UK’s current global land use footprint, and no previous work has explored how this might evolve under future scenarios. We provide an update on the historical land footprint of UK food and feed imports from 2010 to 2020 and produce spatially explicit estimates of the land footprint from 2020 to 2100. Food and feed demand, agricultural production and trade are simulated using a food system model under four global socioeconomic and climate scenarios. Using biophysical accounting, we estimate that 11 Mha of agricultural land is currently linked to UK food and feed imports. Across all scenarios, we estimate that the global land footprint of UK food and feed imports will reach 10–12 Mha of agricultural land by 2050 and 10–16 Mha by 2100. With 17 Mha of UK land currently used for agriculture, the land footprint of food and feed imports should be an important focus when evaluating the environmental consequences of UK food consumption.

## Introduction

The land area associated with the production of agricultural commodities has been termed the land footprint, embodied land, or virtual land [1–3]. Between 1986 and 2016, the cropland footprint associated with global agricultural trade increased from 128 Mha to 350 Mha, representing nearly a third of global arable land use [3]. Including cropland and pasture, approximately 1000 Mha of agricultural land (22% of total agricultural land area) has been recently (2004–2017) linked to traded agricultural products [1]. This highlights the significance of international trade for global land use patterns.

International food trade has previously been linked to negative environmental impacts [1,4–6]. For example, the EU’s increasing demand for biodiesel and animal feed is a major driver of deforestation in tropical countries [4]. Globally, 29–39% of tropical deforestation-related GHG emissions can be linked to agricultural and forestry trade [6]. GHG emissions from agricultural production and forestry embodied in international trade account for 27% of global land use emissions [1]. Of these, approximately 75–81% are due to land conversion, and the remainder are due to GHG emissions from cultivation [1].

Nevertheless, there are considerable differences in the environmental impact of different food groups, and some imported crops have a lower environmental impact than the equivalent produced domestically [7,8]. Linkages between agricultural imports and land use and land cover change (LULCC) are complex and depend on multiple interactions between producers, commodity markets, and consumers [9]. Establishing effective regulatory frameworks to address the environmental consequences of international trade has proven difficult, as illustrated by the EU’s approach to addressing global deforestation [4,5].

Previous studies typically use one of two approaches to estimate land footprints of traded commodities – biophysical accounting and Multi-Region Input Output (MRIO) analysis. Biophysical accounting relies on reported yields and detailed trade tables to calculate the area of land associated with trade flows in each country or region [10]. In contrast, MRIO models link national input-output tables of financial transactions between a country’s economic sectors with tables of international trade flows [10,11]. In an environmentally-extended MRIO, production factors are added to the MRIO framework, allowing for the estimation of the environmental impact (such as the land footprint) of each unit of final demand [11]. MRIO analysis captures the entire supply chain, making it useful for assessing the broader, indirect land footprint of traded commodities. However, it can oversimplify land use impacts by assuming uniformity of production within a sector. In contrast, biophysical accounting can provide spatially detailed information, allowing more direct links to environmental impacts.

Estimates of land footprints can vary considerably due to uncertainties in trade and crop yield data, and methodological differences between studies. For example, estimates of net cropland area embodied in China’s trade range from −17 Mha to 19 Mha, i.e., from a net export to a net import of land [2]. A previous estimate for the UK showed that 11 Mha of the UK’s cropland footprint (70% of the total) was located abroad in 2008 [12]. However, more recent estimates are not available. Given changing global trade patterns, up-to-date estimates and future projections of countries’ land footprints are needed to allow us to better understand and address the environmental impacts of agricultural trade.

The United Kingdom is a net food importer, with imports accounting for 46% of total food consumption [13]. Globally, the UK is the third largest food importer in terms of net trade value, behind China and Japan [14]. Recent global events, such as the COVID-19 pandemic and the war in Ukraine, have exemplified the importance of the interconnectedness of the global food system for food security [15,16]. The UK’s reliance on food imports leaves the country’s food system potentially vulnerable to external impacts such as those arising from climate change, international conflict, and market shocks [13,17]. Furthermore, UK food and feed imports have previously been linked to land use and land use change (LULUC) and greenhouse gas (GHG) emissions abroad [12,18,19]. Regular monitoring of the UK’s global land footprint is required to assess the country’s global environmental impacts and susceptibility to food system shocks.

Here, we estimate the UK’s global land footprint from 2010 to 2020 and, subsequently, explore potential future trajectories up to 2100 using the Land System Modular Model (LandSyMM). We present globally aggregated and mapped estimates of the UK’s land footprint for four distinct scenarios representing a range of global socioeconomic, dietary, and climate outcomes.

## Methods

### Land use modelling framework

**Fig 1:**
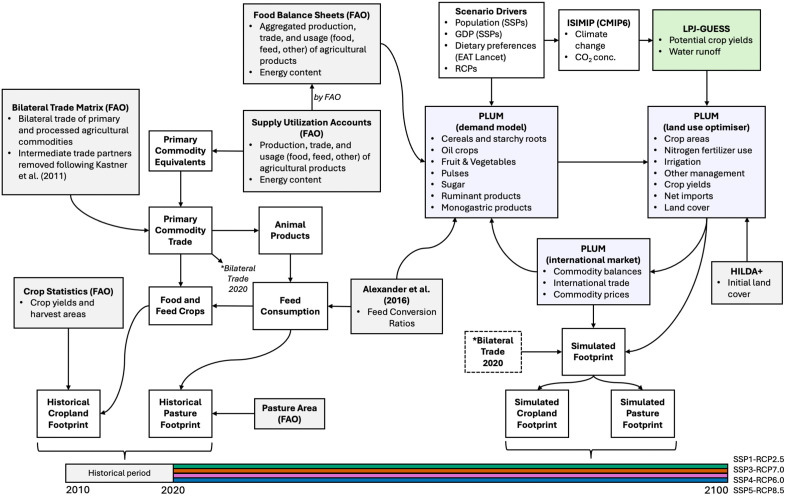
Schematic of key data sources and data processing for the estimation of historical and simulated land footprint of UK food and feed imports.

The Land System Modular Model (LandSyMM; https://landsymm.earth) is a spatially explicit model of the global land system that couples a dynamic global vegetation model (LPJ-GUESS) and a land use model (PLUM). LandSyMM simulates country-level food demand, international trade of agricultural commodities, spatially explicit yields, and food and energy crop production. Here, we use LandSyMM to investigate the UK’s global land footprint for a range of future socioeconomic and climate scenarios. We use a biophysical accounting approach for calculating land footprints by directly linking simulated land use to estimated trade flows (Fig 1).

The Lund-Potsdam-Jena General Ecosystem Simulator (LPJ-GUESS) is a process-based dynamic global vegetation model which simulates ecosystem processes including vegetation and soil carbon dynamics, the nitrogen cycle, and plant physiological responses to climate change, atmospheric CO_2_, and disturbance [20,21]. LPJ-GUESS can simulate crop yield responses to changes in atmospheric CO_2_ levels and irrigation intensity as well as nitrogen management, deposition and biological fixation [22,23]. Here, we used LPJ-GUESS version 4.1 to generate potential yield responses for 10 crop functional types (CFTs) and pasture under different climate, nitrogen fertilisation, and irrigation regimes. A second-generation bioenergy crop (*Miscanthus*) was modelled as a C4 cereal CFT. We used climate data from the Inter-sectoral Impact Model Intercomparison Project (ISIMIP), bias-corrected Coupled Model Intercomparison Project Phase 6 (CMIP6) for the MRI-ESM2–0 general circulation model [24,25]. Simulated yields were calibrated to observed country average yields for the period 1995–2005, accounting for nitrogen fertiliser use, irrigation, and GDP per capita as a proxy for other inputs such as pesticide use and mechanisation (S1 Fig in S1 File).

The Parsimonious Land Use Model (PLUM) simulates land use and land use change from changes in demand for food commodities and bioenergy, changes in crop yields, and international trade [26]. Food demand is projected using a Modified An Implicit Directly Additive Demand System (MAIDADS) [27,28] which uses per capita income (exogenous) and commodity prices (modelled endogenously in PLUM) to calculate demand for seven food groups (S2 Fig in S1 File). Regional demand for second generation bioenergy was taken from the IIASA SSP Database [29] and disaggregated to country level based on potential *Miscanthus* production in the baseline year (2020) using yields from LPJ-GUESS. However, countries are not constrained to produce the prescribed energy crop demand and may import or export any shortfall or excess. The initial land cover distribution was taken from HILDA+ and mapped to PLUM land cover classes [30]. The initial crop area distribution is from SPAM2010 [31].

We used population and GDP (2017 PPP) projections from Koch and Leimbach [32] who harmonise and update projections from the IIASA SSP database [29] with recent demographic and economic changes (S4 and S5 Figs in S1 File). Changes in global dietary preferences were modelled as a shift from historical dietary patterns towards a healthier and more sustainable diet based on the EAT Lancet recommendation [33]. The degree of shift in dietary preferences was determined by the authors’ interpretation of the scenario narratives with a complete shift in SSP1, an intermediate shift in SSP4, and no change in preferences in SSP3 and SSP5. Changes in dietary preferences were modelled for all countries, not just for the UK (see text in S1 File).

During each time step, PLUM uses least-cost optimisation to determine land use factors including cropland and pasture area, fertilizer input, irrigation, and a management intensity factor (representing, for example, pesticide use, phosphorus fertiliser and mechanisation). Crop yields are interpolated for a continuous range of fertilizer application rates and irrigation using yield tables generated by factorial experiments in LPJ-GUESS. Pasture yield responses to fertilisation and irrigation are not modelled here. Irrigation is constrained at the water basin level by the estimated surface water runoff modelled by LPJ-GUESS. PLUM is constrained to produce sufficient food and bioenergy to meet demand, through domestic production or imports. A single international market allows countries in PLUM to import and export commodities whose prices are adjusted on an annual basis based on the net balance of imports and exports. Bilateral trading is not currently modelled in PLUM. To simulate uncertainty in modelled outcomes, simulations were repeated with stochastically sampled input parameters from distributions consistent with scenario narratives (S3 Table in S1 File).

LandSyMM has been developed as a global land system model and its structural validity primarily operates at that scale. The model captures global trade dynamics, aggregate land use change, and cross-country feedbacks between food demand, agricultural production and commodity markets. While the UK’s land footprint is inherently determined by global dynamics, we acknowledge that country-specific outputs present greater uncertainty than globally aggregated results. As such, our results should be interpreted primarily in terms of directional trends and relative differences between scenarios, rather than high-precision forecasts. Nevertheless, the model performs well in reproducing long-term historical land use patterns [26] and is therefore suitable for global-scale future projections. LandSyMM is not constrained to reproduce baseline data exactly, instead land use patterns emerge endogenously through modelling of land use decision making. This process-based approach allows the model to respond dynamically to changes in demand, trade balances and environmental conditions. A country-wise comparison for 2020 shows high agreement between the simulated and reported distribution of crop areas and production (S11 and S12 Figs in S1 File).

### Calculation of trade flows

We calculated the historical land footprint of UK food and feed imports by combining reported trade, production and consumption of food and feed commodities from FAOSTAT [34]. We used methods from Kastner et al. [35] to establish each traded commodity’s production origin from reported bilateral trade data. This step is necessary to remove intermediate trade partners which import and then re-export commodities (for example, the Netherlands is a major exporter of bananas in Europe). For historical trade flows, we used bilateral trade data (Detailed Trade Matrix) from FAOSTAT [34]. The method detailed by Kastner et al. [35] transforms the reported bilateral trade matrix Z into matrix R, which removes intermediate trade partners, therefore showing only trade flows between producers and consumers. The following calculation steps are summarised from Kastner et al. [35]:


$$\begin{array}{c} 𝐱=𝐩+𝐙\cdot 𝐢, \end{array}$$ (1)

where 𝐱 is a vector of total domestic material input (production plus imports; DMI), 𝐩 is the production vector, 𝐙 is the bilateral trade matrix (such that $z_{ij}$ is the import by country i from country j), and 𝐢 is a vector of ones.


$$\begin{array}{c} 𝐀=𝐙\cdot {\hat{𝐱}}^{-\text{1}}, \end{array}$$ (2)

where 𝐀 is a matrix of export shares, $\hat{𝐱}$ is a matrix with 𝐱 on the diagonal and zeroes elsewhere.


$$\begin{array}{c} 𝐑={\left(𝐈-𝐀\right)}^{-\text{1}}\cdot \hat{𝐩}, \end{array}$$ (3)

where 𝐑 is the matrix in which $r_{ij}$ is part of the DMI of country i that is produced in country j, 𝐈 is the identity matrix, and $\hat{𝐩}$ is a matrix with 𝐩 on the diagonal and zeroes elsewhere. The trade flows in matrix 𝐑 include both primary crops and animal commodities (e.g., wheat and milk) and secondary products (e.g., flour and cheese) produced from primary commodities.

### Calculation of primary crop and animal product equivalents

Traded commodities were converted to primary crop equivalents or primary animal product equivalents using a method developed here, which links the processing of primary products to the production of processed secondary products. For each country, we used commodity processing trees from the FAO [36] and non-linear optimisation to allocate crop and animal products used in processing to their resultant products, assuming conservation of the energy content and allowing for losses. We calculated conversion factors using the estimated processing flows between secondary products and primary crop or animal products. The conversion factors calculated here consider all potential inputs and outputs of commodities and, therefore, avoid double counting by allocating primary products to secondary products proportionally by energy content. Using these conversion factors and the corrected bilateral trade matrix, we obtained bilateral trade flows of food and feed commodities expressed in primary crop or animal product equivalents. We assumed that exported secondary products were produced proportionally from domestic and imported primary products.

We used reported utilisation of primary commodities for processing (the inputs) and production of secondary products (the outputs) from the Supply Utilization Accounts (SUA) [34] and commodity processing trees [36] to derive conversion factors between input and output commodities. Conversion factors were calculated using non-linear optimisation by allocating inputs to the production of outputs:

*Minimise:*


$$\begin{array}{c} \sum_{\text{j}}{\text{w}}_{\text{j}}{\;(\;(\sum_{\text{i }\in \text{P}(\text{j})}{\text{a}}_{\text{i},\text{j}}k_{i}\;)-\;{\text{p}}_{\text{j}}\;{\text{k}}_{\text{j}}\;)}^{\text{2}}+\text{ 100 }\sum_{\text{i}}{\;({\text{r}}_{\text{i}}\;{\text{k}}_{\text{i}}\;)}^{\text{2}}; \end{array}$$ (4)

$$\begin{array}{c} {\text{w}}_{\text{j}}=\;\frac{\text{1}}{{\text{p}}_{\text{j}}\;{\text{k}}_{\text{j}}}; \end{array}$$ (5)

*subject to:*


$$\begin{array}{c} \;(\sum_{\text{j }\in \text{Q}(\text{i})}{\text{a}}_{\text{i},\text{j}}\;)+\;{\text{r}}_{\text{i}}=\;{\text{q}}_{\text{i}}\;for\;each\;i; \end{array}$$ (6)

$$\begin{array}{c} \sum_{\text{i }\in \text{P}(\text{j})}{{\text{a}}_{\text{i},\text{j}}\text{k}}_{\text{i}}\leq \text{1}.\text{25 }{\text{p}}_{\text{j}}\;{\text{k}}_{\text{j}}\;for\;each\;j, \end{array}$$ (7)

where ${\text{a}}_{\text{i},\text{j}}$ is the allocation of input i to output j, k is the energy content, p is the produced output amount, q is the processed input amount, r is the residual amount of inputs that could not be allocated to production, P(j) returns the set of all inputs i which can produce j, and Q(i) returns the set of all outputs j which can be produced from input i. Equation 4 defines the weighted sum of squared differences between the energy content of output j and all the inputs i plus a penalty for unallocated residual inputs. Squared differences are weighted by ${\text{w}}_{\text{j}}$ (Equation 5) so that any inputs above what is required to meet the energy contents of the outputs are distributed proportionally by the energy content of the output, therefore implying a constant waste ratio across outputs. The residual unallocated penalty is weighted by 100 to favour allocation to outputs. Equation 6 is a constraint such that all inputs are accounted for. Equation 7 constrains the conversion factors such that the ratio of the energy content of inputs is no more than 1.25 times the energy content of outputs (to allow for conversion losses). The energy content of each food item is derived from the SUA dataset and supplemented with values from FAO Food Composition Tables [37].

We used these conversion factors to convert all trade flows in matrix 𝐑 into primary crop or animal product equivalents. We assumed that secondary products are produced from both domestically produced and imported primary products, with the amount of input from domestic production and imports being proportional to their sizes. The imported commodities were then traced to their country of production using matrix 𝐑. The procedure was repeated if further conversion steps were needed until all amounts were in primary crop or animal product equivalents.

### Calculation of historical land footprints

Imports were split between food, feed, and other uses (waste, seed and other), assuming the same allocation as for the entire supply. Other uses were ignored in all analyses. For crops, trade flows were converted to land footprints by dividing the amount of traded commodity by the reported yield in the producing country:


$$\begin{array}{c} \text{land footprint}=\;\frac{\text{crop amount}}{\text{crop yield}} \end{array}$$ (8)

We used a modified method from Alexander et al. [38] to allocate cropland used for feed production and pasture to the production of animal products. The land footprint of animal products includes both a feed crop component and a pasture component. We calculated embodied feed crops in animal products by first estimating the feed requirements of each animal product. We used feed conversion ratios (FCRs) from Alexander et al. [38], estimated for poultry, pork, beef, other meat, eggs, and whole milk. These FCRs represent the feed requirement per unit of edible weight, including by-products such as animal fats and offal, which are reported separately in the FAO data. To obtain separate FCRs for meat, animal fats, and offal, we disaggregated the base FCRs for each group of animal products using extraction rates from FAO Commodity Trees [36] and the energy content of the items:


$$\begin{array}{c} {\text{f}}_{\text{i}}={\text{f}}_{\text{base}}\frac{{\text{w}}_{\text{i}}\cdot {\text{k}}_{\text{i}}}{\sum_{\text{j}}{\text{w}}_{\text{j}}\cdot {\text{k}}_{\text{j}}} \end{array}$$ (9)

where ${\text{f}}_{\text{i}}$ is the FCR of item i, ${\text{f}}_{\text{base}}$ is the base FCR from Alexander et al. [38], ${\text{w}}_{\text{i}}$ is the extraction rate, and ${\text{k}}_{\text{i}}$ is the energy content.

Reported feed usage was converted to dry-matter equivalents using dry-matter ratios from INRAE et al. [39]. The reported feed usage in each country was then allocated to animal products proportionally by the feed requirement of each animal product. Feed was first allocated to monogastrics (pigs, poultry, and eggs) and then to ruminants (cattle, milk, sheep, goats, and others). Finally, feed usage was converted to a land footprint in the same way as for food crops (Equation 8), tracing any feed imports to producers.

The pasture footprint of ruminant products was calculated by allocating pasture area proportionally by estimated feed requirement [38]. We assumed that all reported permanent pasture area was used for ruminant production. This approach can produce inflated pasture footprints in countries where the ratio of pasture area to total ruminant production is anomalously large. This can arise if the reported pasture area is not fully utilised or due to ambiguities around land cover classification. For this reason, we excluded Namibia and Botswana from our estimations of pasture footprints.

### Calculation of simulated future land footprints

We calculated future land footprints using a biophysical accounting approach where trade flows are linked directly to land use. Crop and pasture areas, feed usage, and net trade flows were simulated explicitly in PLUM. To estimate gross imports, we took reported import levels from FAOSTAT [34] for the baseline year (2020) and projected these based on changes in net imports and domestic production predicted by PLUM. We assumed that the import share of supply changes linearly with the self-sufficiency ratio (production divided by supply) based on a relationship derived from historical data (S6 Fig in S1 File).


$$\begin{array}{c} {\text{m}}_{\text{i}}={\text{s}}_{\text{i}}\cdot {\text{r}}_{\text{i}}; \end{array}$$ (10)

$$\begin{array}{c} {\text{r}}_{\text{i}}=\text{max}\{{\text{b}}_{\text{i}}-\text{0}.\text{8}\cdot {\text{z}}_{\text{i}},\text{0}.\text{2}\}; \end{array}$$ (11)

$$\begin{array}{c} {\text{z}}_{\text{i}}=\frac{{\text{p}}_{\text{i}}}{{\text{s}}_{\text{i}}}, \end{array}$$ (12)

where ${\text{m}}_{\text{i}}$ is the import of product i, ${\text{s}}_{\text{i}}$ is the supply, ${\text{r}}_{\text{i}}$ is the import ratio, ${\text{z}}_{\text{i}}$ is the self-sufficiency ratio and ${\text{p}}_{\text{i}}$ is the production. Parameter b was calculated such that imports derived from simulated production and supply matched the reported imports in the baseline year:


$$\begin{array}{c} {\text{b}}_{\text{i}}=\overset{\text{rep}}{\underset{\text{i}}{\text{r}}}+\text{0}.\text{8}\cdot \overset{\text{base}}{\underset{\text{i}}{\text{z}}}, \end{array}$$ (13)

where $\overset{\text{rep}}{\underset{\text{i}}{\text{r}}}$ is the reported import ratio in 2020 from FAOSTAT and $\overset{\text{base}}{\underset{\text{i}}{\text{z}}}$ is the baseline self-sufficiency ratio in 2020 from PLUM. We assumed that the import ratio $r_{i}$ does not go below 20%, which is in line with reported historical data for the UK (S6 Fig in S1 File). This accounts for imports of crops and products which cannot be produced in the UK (e.g., tropical fruit) and demand for non-UK products.

As PLUM does not model bilateral trade, trade between countries and the international market needed to be disaggregated so that imported commodities could be traced to their country of origin. We used the derived bilateral trade matrix from above, averaging trade over three years (2018–2020) to reduce the impact of annual trade variations. We assumed that trade preferences remain constant through time – i.e., for each importing country, the share of each commodity imported from an exporting country remains constant. In cases where exports exceeded 35% of domestic production (based on maximum historical export shares), excess exports were reallocated to other countries with additional export capacity proportionally by the size of their exports to the UK. This method represents the observed desire for exporting countries to maintain diverse trading relationships and ensures that export shares can readjust if production in the exporting country declines.

To calculate the land footprints of traded commodities in PLUM, we applied an equivalent procedure to that used when calculating historical land footprints. Demand, trade and production of crops and animal products in PLUM are already expressed in primary crop or feed equivalents and, therefore, do not need to be converted as for the historical data. Feed usage (for both monogastrics and ruminants) is also simulated explicitly in PLUM, although this can differ from observed usage given that the model is not constrained to reproduce baseline conditions. To obtain better estimates of the UK’s feed usage, we rebased simulated feed usage to match observed usage in the baseline year. We assumed that total feed usage (in dry-matter equivalent mass) was as predicted by the model, but the split between different feed crops matched the reported data. Simulated trade flows of food and feed commodities were converted to land footprints by assuming that the production of exported commodities within each country was evenly distributed over the production area, weighted proportionally by production quantity.

### Description of future scenarios

We report projections of the land footprint of UK food and feed imports from 2020 to 2100 for four global SSP-RCP scenarios (Shared Socioeconomic Pathways; Representative Concentration Pathways): SSP1-RCP2.6, SSP3-RCP7.0, SSP4-RCP6.0, and SSP5-RCP8.5; (hereafter SSP1–5; Table 1). These scenarios were chosen to represent a range of potential future socioeconomic and climate trajectories with contrasting population and economic growth levels – the key factors driving food demand in LandSyMM. We did not include SSP2 as the socioeconomic narrative of this scenario falls broadly between the other four, therefore contributing little to the range of outcomes presented here.

**Table 1:** Description of the global socioeconomic scenarios explored in this study. Narratives are summarised from O’Neill et al. [40]. Changes in dietary preferences towards the EAT Lancet diet [33] are based on the authors’ interpretation of scenario narratives.

Scenario	Socioeconomic narrative	Dietary preferences
SSP1-RCP2.6	A shift towards sustainability characterised by a focus on environmental protection, investment in education, health, and international cooperation. Reduced consumption leads to reduced pressure on the environment and lower GHG emissions. Population growth is low. High economic growth results in a rapid increase in average incomes.	Rapid reduction in the consumption of animal products and sugar, and an increase in the consumption of pulses and oil crops. All countries converge towards a healthier, more sustainable diet as incomes increase. Dietary preferences shift 100% towards the EAT Lancet diet by 2050.
SSP3-RCP7.0	Lack of international cooperation, regional conflict and weak global institutions lead to a lack of concerted effort in addressing environmental and societal problems. Resource use and fossil fuel intensity are high. Population growth is uneven, with higher growth in developing countries. Economic growth is sluggish and little progress made in developing countries.	Dietary preferences remain unchanged from historical patterns characterised by growing consumption of animal products, oil crops, and sugar as incomes increase.
SSP4-RCP6.0	Global disparities in economic prosperity grow due to uneven investment in human capital. A shift towards sustainability and reducing GHG emissions is mostly limited to high income countries with little progress elsewhere. Population growth is low in high-income countries and high in low-income countries. There is moderate economic growth in industrialised and middle-income countries and low growth in low-income countries.	Unequal socioeconomic development leads to a partial progress towards healthier and more sustainable diets. Dietary preferences shift 50% towards the EAT Lancet diet by 2050.
SSP5-RCP8.5	Fossil-fuel-driven development results in relative prosperity and rapid development globally. The focus on economic growth means that global environmental issues are neglected and GHG emissions are high. Demand for commodities is high due to rapid economic growth. Population growth is low and rapid economic growth leads to a very high global average income.	Dietary preferences remain unchanged from historical patterns characterised by growing consumption of animal products, oil crops, and sugar as incomes increase.

**Fig 2:**
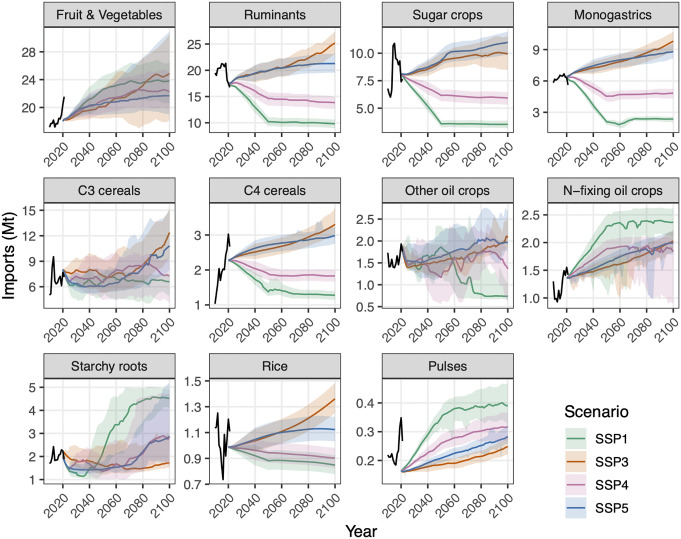
Simulated UK imports of food and feed commodities from 2020 to 2100 under each scenario. Crops are represented in primary crop equivalents and animal products (ruminants and monogastrics) are in dry-matter feed equivalents. Solid lines show median values and shaded areas represent 90% uncertainty ranges from a stochastic sampling of model parameters (n = 30 ensemble members for each scenario). Historical imports are shown for 2010-2020 by solid black lines.

We modelled changes in global dietary preferences (including but not restricted to the UK) as a shift from historical patterns towards a diet based on the EAT Lancet recommendation (Willett et al. 2019). In the UK, a shift in dietary preferences results in a large reduction in the imports of sugar and animal products in SSP1, with smaller decreases in SSP4 (Fig 2). In SSP3 and SSP5, imports of most food and feed commodities increase from present levels. Changes in imports are strongly influenced by domestic demand (S7 and S8 Figs in S1 File) driven by population and economic growth (S4 and S5 Figs in S1 File). However, other factors including commodity prices (S9 Fig in S1 File), changes in agricultural productivity in the UK, and scenario parameterisation can lead to import patterns that diverge from demand. For example, while per capita demand for fruit and vegetables is lowest in SSP3, imports are higher than in other scenarios due to a combination of high population growth and low food prices.

## Results

### Historical land footprint

**Fig 3:**
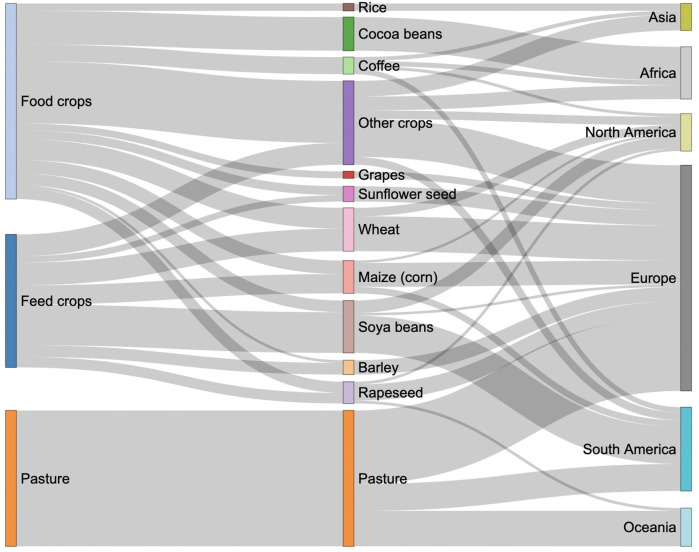
Sankey diagram illustrating the land footprint of UK food and feed imports by usage (left column), primary crop (centre column), and production region (right column) in 2020. The width of nodes and links is proportional to the size of the land footprint. The top ten crops with the largest footprint and pasture are shown individually, and the remainder are grouped as “Other crops”. For visual clarity, only trade flows of at least 0.05 Mha are shown, representing 94% of the total land footprint. Data were derived from FAOSTAT [34] and regional groupings are according to Our World in Data [42].

Using reported data from the FAO [34], we estimate that 7.8 Mha of cropland and 3.0 Mha of pasture were linked to UK food and feed imports in 2020 (Fig 3). Of the total cropland footprint, 4.7 Mha was from food production and 3.1 Mha from feed production (1.5 Mha from imported feed and 1.6 Mha from feed embodied in imported animal products). Added together, the total agricultural land footprint of UK food and feed imports in 2020 was 10.8 Mha, an area that is over half of the UK’s agricultural area (16.8 Mha) [41]. Just five crops (soya beans, wheat, cocoa beans, maize, and rapeseed) contributed 55% of the cropland footprint. Of these, the largest contributors were soya beans (1.2 Mha), wheat (1.0 Mha), and cocoa beans (0.78 Mha) (S4 Table in S1 File).

**Fig 4:**
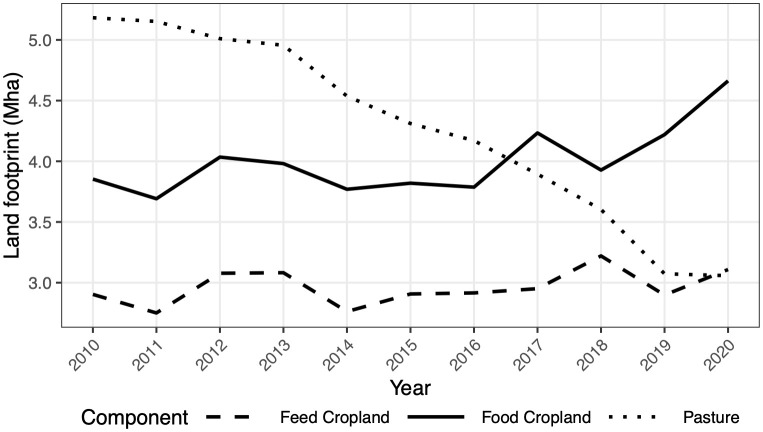
Historical land footprint of UK food and feed imports from 2010 to 2020. The feed cropland footprint includes both direct feed imports and feed embodied in imported animal products. Data were derived from FAOSTAT [34].

Furthermore, our estimates suggest that the UK’s global cropland footprint has increased by 15% between 2010 and 2020, while the pasture footprint has decreased by 41% (Fig 4). The steep decline in the pasture footprint can be explained by the intensification of ruminant production in exporting countries. For example, ruminant production in Ireland (a major exporter to the UK) increased by 56% from 2010 to 2020 despite virtually no change in the reported pasture area [34]. Given our assumption that all reported pasture area is utilised for ruminant production, increasing ruminant production in a stable pasture area will result in a lower footprint for each unit of produce.

The global distribution of the cropland footprint of the UK’s food and feed imports is highly concentrated in Europe, accounting for 44% (3.6 Mha) of the footprint (Fig 3). Of the remainder, 17% (1.4 Mha) was in South America, 15% (1.2 Mha) in Africa, 11% in North America (0.90 Mha), 10% in Asia (0.85 Mha), and 3% in Oceania (0.20 Mha). The UK’s pasture footprint distribution shows a similar concentration in Europe and South America but with a considerably larger share in Oceania. Europe accounts for 53% (1.6 Mha) of the UK’s global pasture footprint, with a further 25% (0.78 Mha) located in Oceania, 19% in South America (0.59 Mha), and the remaining 3% in the rest of the world.

### Simulated land footprint

**Fig 5:**
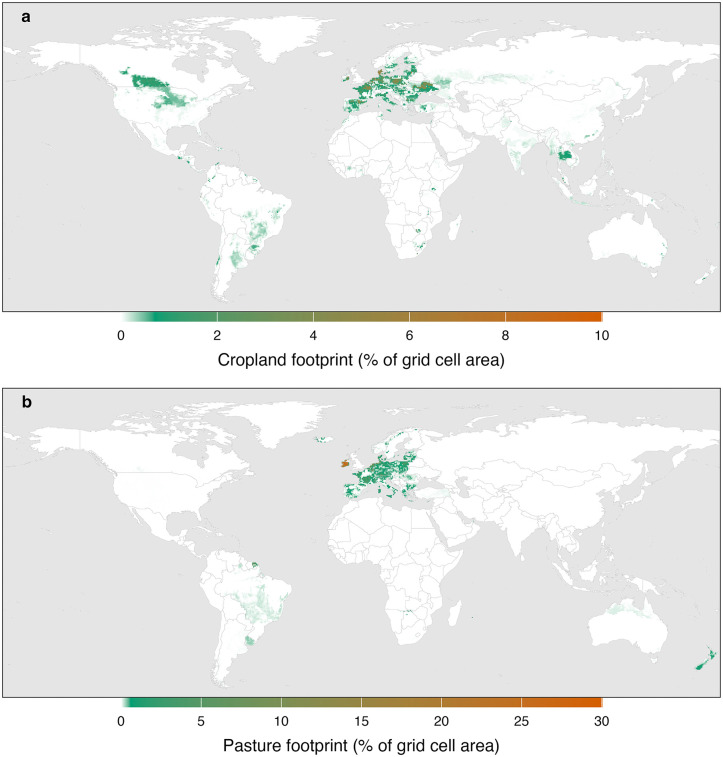
Simulated spatial distribution of the global (a) cropland and (b) pasture footprint of UK food and feed imports in 2020. Land footprints are represented as a percentage of the total grid cell area. Made with Natural Earth (https://www.naturalearthdata.com).

Using LandSyMM, we simulated the spatial distribution of the UK’s global cropland and pasture footprints in 2020 (Fig 5). The simulated total agricultural land footprint (11.7 Mha) is broadly compatible with our estimate from reported FAO data, albeit with a lower cropland footprint (6.3 Mha) and a higher pasture footprint (5.4 Mha). The simulations also show a higher concentration of the UK’s land footprint in Europe (63% for cropland and 96% for pasture) due to differences between simulated and observed crop yields and pasture usage.

The land footprint of UK food and feed imports is highly dispersed, extending over most of Europe and large parts of the Americas. While the footprint within individual grid cells can reach as high as 30% of the grid cell area for pasture and 10% for cropland, the UK’s footprint is below 1% in the vast majority of grid cells. It’s important to highlight that these estimates assume that the land footprint of exported commodities is distributed uniformly over the production area within each country. In reality, the production of exported commodities could be more concentrated (for example, in the most profitable regions or locations with better transport links), creating a different land footprint distribution than domestically consumed commodities. Despite this limitation, our results provide an approximation of the UK’s agricultural land footprint, which can be further refined by incorporating more spatially detailed information about global trade patterns.

### Scenario results

**Fig 6:**
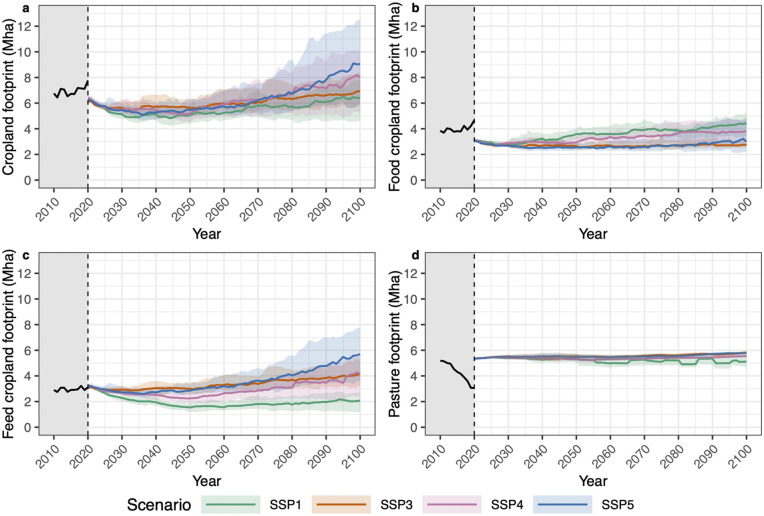
The global land footprint of UK food and feed imports in terms of (a) total cropland area, (b) cropland area for food production, (c) cropland area for feed production (both imported feed and feed embodied in imported animal products), and (d) pasture area. Solid lines show median values and shaded areas represent 90% uncertainty ranges from a stochastic sampling of model parameters (n = 30 ensemble members for each scenario). Historical land footprints are shown for 2010-2020 (grey shaded area) and simulated footprints for 2020-2100.

Results suggest that the global land footprint of UK food and feed imports will remain broadly comparable to present levels throughout the 21^st^ century (Fig 6). Across all scenarios, median estimates of the total agricultural land footprint are 11.6 Mha in 2020, 10.7 Mha (90% uncertainty range: 10.1–11.9) in 2050 and 12.8 Mha (10.5–16.7) in 2100. The simulated land footprints show some bias compared to historical estimates, particularly for pasture and cropland used for food production. The simulated total agricultural footprint is 8% higher, the cropland footprint is 19% lower, and the pasture footprint is 80% higher than estimates from FAO data. During the initial model spin-up, PLUM is not constrained to reproduce baseline conditions and is allowed to reach an equilibrium state. This ensures that land system changes in future scenario simulations result from scenario drivers (e.g., population, incomes, yields) rather than internal model dynamics. As a result, trade levels, demand, prices, and land use do not reproduce precisely the initial observed conditions, leading to an offset in simulated results. Despite this, we find strong country-wise agreement between simulated land footprints and those estimated from historical data with a Pearson correlation coefficient of 0.70 for cropland, 0.75 for feed crops, 0.68 for food crops and 0.72 for pasture (S13 and S14 Figs in S1 File). Nevertheless, absolute values of simulated footprints should be interpreted with caution and conclusions should primarily be drawn from relative changes.

Scenarios with a shift in dietary preferences towards the EAT Lancet diet (SSP1 and SSP4) show a decrease in the total cropland footprint until 2040−2050, driven by a reduction in feed and animal product imports. By 2050, the cropland footprint of feed imports (including feed embodied in animal products) decreases considerably in these two scenarios, with a median change of −51% (90% uncertainty range: −62% to −40%) in SSP1 and −32% (−44% to −20%) in SSP4 (Fig 6C). There is little change in SSP3 (−3%; −16% to 14%) and SSP5 (−12%; −22% to 1%), where historical patterns in dietary preferences are maintained. From 2050 to 2100, the cropland footprint of feed imports remains stable in SSP1 but increases in SSP3, SSP4 and SSP5, reaching similar or higher levels to the present day. The cropland footprint of food imports shows a contrasting trend, increasing by 43% (90% CI: 11% to 70%) by 2100 in SSP1 and by 20% (−14% to 53%) in SSP4, with a small decrease in SSP3 of −10% (−16% to 6%) and −2% (−28% to 59%) in SSP5 (Fig 6B).

The contrasting trends in the cropland footprints of food and feed imports are primarily explained by differences in agricultural intensity between scenarios (S10 Fig in S1 File). Land footprints are a product of both consumption level and agricultural intensity. Therefore, both factors must be considered to understand how the UK’s global land footprint evolves under each scenario. In both SSP3 and SSP5, greater imports are balanced by rapid increases in global agricultural intensity. This increase in intensity occurs for contrasting reasons which drive food demand: in SSP3 due to rapid population growth and in SSP5 due to rapid income growth and scenario parameterisation which favours intensification. As a result, despite higher food and feed imports in SSP3 and SSP5, the total land footprint remains comparable to other scenarios. The patterns of agricultural intensification and expansion across the scenarios have a significant impact of the UK’s import land footprint and therefore trends in the simulated footprint may not entirely mirror the trends in import levels.

Despite a 41% reduction in imports of ruminant products in SSP1 and a 15% increase in SSP3 by 2050, we only observe minor differences in pasture footprints between the scenarios (Fig 6D). There is a slight increase in the pasture footprint of 8% (90% uncertainty range: 5% to 11%) in SSP3, 8% (4% to 11%) in SSP5, and 3% (2% to 5%) in SSP4. In contrast, SSP1 shows a small decrease of −4% (−12% to 4%). In scenarios with a shift in dietary preferences (SSP1 and SSP4), a lower demand for ruminant products (both globally and in the UK) is accompanied by a reduction in pasture use intensity. As a result, each unit of imported animal product is associated with a higher land footprint, which offsets the effect of a reduction in imports.

**Fig 7:**
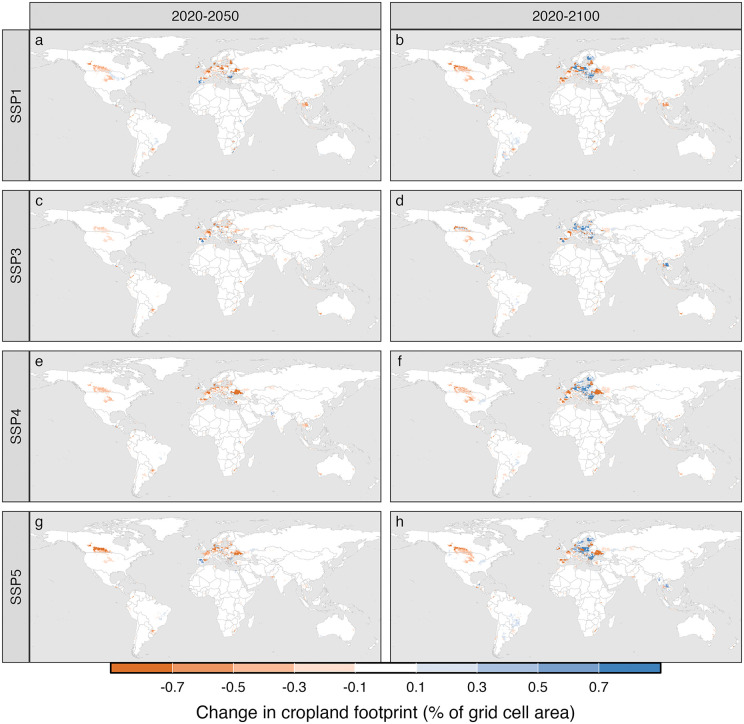
Spatial distribution of changes in the cropland footprint of UK food and feed imports by SSP (panel rows) for 2020-2050 (a, c, e, g) and 2020-2100 (b, d, f, h). Made with Natural Earth (https://www.naturalearthdata.com).

**Fig 8:**
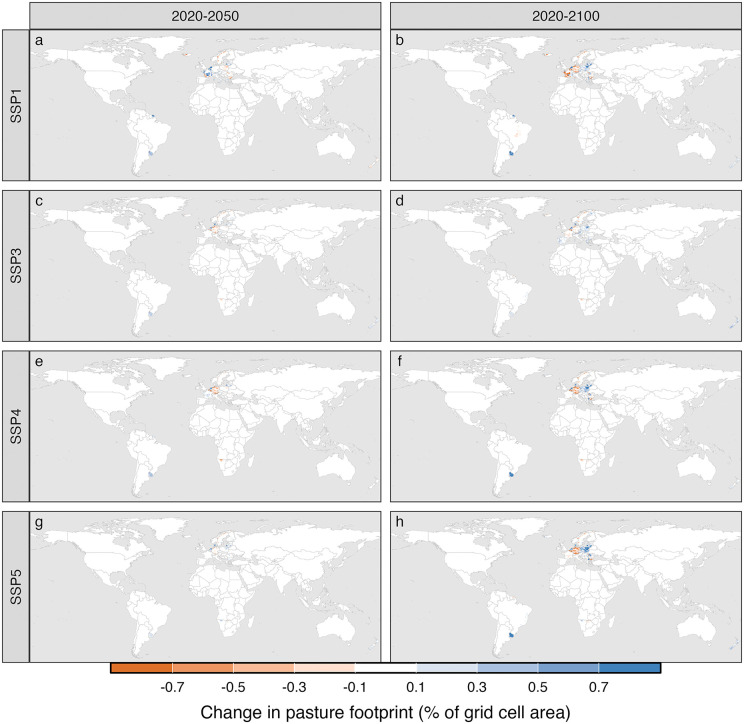
Spatial distribution of changes in the pasture footprint of UK food and feed imports by SSP (panel rows) for 2020-2050 (a, c, e, g) and 2020-2100 (b, d, f, h). Made with Natural Earth (https://www.naturalearthdata.com).

Aggregated global trends in the land footprint of UK food and feed imports mask some important local and regional differences. Spatial changes in the UK’s global land footprint are not uniform but differ considerably between regions and within individual countries (Figs 7 and 8). For example, while there is an overall decrease in the UK’s cropland footprint in Europe from 2020 to 2050, in most scenarios, parts of southern Europe see an increase in the cropland footprint driven by growing imports of fruit and vegetables. The spatial pattern of changes in the UK’s global land footprint from 2020 to 2050 is broadly similar across all scenarios but diverges towards the end of the century. Individual countries show areas of both increasing and decreasing footprints driven by changes in the composition of the UK’s imports, the distribution of crop production, and the exporting countries’ supply balances.

## Discussion

To our knowledge, this study is the first to evaluate the land footprint of UK food and feed imports under a range of future socioeconomic scenarios. In addition, we provide up-to-date estimates of the UK’s global land footprint using reported data. We estimate that the total agricultural land footprint of UK food and feed imports has remained relatively stable between 2010 and 2020, falling slightly from 11.9 Mha to 10.8 Mha. However, the overall change conceals contrasting trends in the cropland and pasture footprints. Simulations using LandSyMM suggest that the UK’s total global land footprint will remain similar to present levels, with a median estimate across all scenarios of 10.7 Mha in 2050 and 12.8 Mha in 2100. However, there are considerable differences between scenarios in the total land footprint’s individual components – food, feed, and pasture. These differences are explained by changes in the volume and composition of the UK’s food and feed imports and shifts in the global distribution and intensity of agricultural production.

We find that changes in the future distribution of the UK’s global land footprint could vary considerably between countries and broader regions. Countries react differently to changes in global trade balances due to complex factors including consumer demand, agricultural subsidies, trade barriers, and yield potentials [9]. In LandSyMM, trade between countries is mediated by a single international commodity market without explicit bilateral trading. The simulations presented here assume that future trade preferences remain fixed at current values. While explicit simulation of bilateral trade could lead to different patterns of land use, future bilateral trade policies are difficult to determine and highly uncertain. Future work could improve on this aspect of the study by making explicit predictions about changing bilaterial trade patterns, for example, by incorporating a gravity trade model. While changes in trade preferences due to macroeconomic factors may be in some way predictable, those relating to political factors are not foreseeable. The long temporal scope of the simulations (2020–2100) means that that incorporating potential changes to global trade patterns due to recent geopolitical factors would also require predicting how long those factors may be relevant. To avoid adding further assumptions, we take a parsimonious approach by assuming that future trade patterns are likely to reflect present trade patterns.

Changes in income distributions, dietary shifts, and population growth are key factors determining food trade globally [3]. The land footprint of traded commodities has been linked to factors such as affluence, with high-income countries displacing a larger proportion of their land use abroad [3,43]. LandSyMM explicitly incorporates interactions between trade flows, domestic production, commodity markets, and consumer demand, which allows us to produce more dynamic and realistic projections of food demand, land use, and trade. Given the complexity of the global food system, modelling these interactions is important when exploring future changes in the land footprint of trade flows.

A limitation of the approaches used to estimate land footprints, including the one used here, is that they ignore marginal yield effects and the spatial explicitness of land footprints by averaging yields at national or higher levels [44]. Each additional unit of production may require marginally greater inputs (land, fertiliser, irrigation, etc.); therefore, the land footprint of exported commodities may differ from that of domestic consumption. Conversely, a growing food export market can lead to agricultural intensification, thereby reducing the land footprint of exported commodities. The impact of a country’s trade patterns on global land use depends on the global context and cannot be studied in isolation. Further research is needed to establish the importance of marginal yield effects and market-land use interactions when estimating land footprints.

Much of the focus within UK agricultural policy has been on reducing the environmental impacts of domestic food production, while less attention has been paid to food imports. Since leaving the EU’s Common Agricultural Policy (CAP), the UK has introduced a new legislative framework (The Agriculture Act 2020) to support farmers to produce “public goods” such as improvements in environmental standards and animal welfare [45]. The ability of the UK to enact its agricultural policy also paves the way for the environmental impact of food imports to be reduced. While imports account for nearly half of UK food consumption, 64% of cropland-related GHG emissions associated with the UK’s food supply are located abroad [18]. The environmental impact of UK imports depends on the characteristics of exporting countries, including environmental standards and biodiversity, which are not explored here. A large proportion of the UK’s food and feed imports originate from the tropics, where the production of commodities such as soya beans and palm oil has been linked to increasing pressure on the environment [6,46].

Reducing the UK’s reliance on imports also comes with trade-offs due to competition for land between food production, timber production and nature conservation. The UK is highly reliant on imports to meet its timber demand, with imports accounting for 81% of the timber supply [47]. Plans to increase the domestic production of timber raise concerns about the potential displacement of food production overseas [48]. The UK’s Net Zero Strategy makes special provisions to support low-carbon farming practices, increase tree planting and restore carbon-rich habitats such as peatland [49]. However, there is insufficient analysis of how these land-based mitigation measures could impact the UK’s overall food supply balance and GHG emissions.

Despite links to numerous environmental impacts, international trade remains an important factor in global food security [4,6,50]. Less than a third of the world’s population can meet their demand for major crops from local production within 100 km, highlighting the importance of trade for food accessibility and security [51]. While reliance on imports can leave countries exposed to global supply shocks, in the long-term, greater integration across international markets can also mitigate food security risks such as price volatility caused by climatic extremes [52,53]. International trade may also reduce environmental pressures in biodiversity hotspots by allowing local food demand to be met by imports from countries with lower biodiversity rather than through domestic production [54].

The impact of food consumed in the UK reaches far beyond the UK’s borders. Our results suggest that the global land footprint of UK food and feed imports will remain a substantial component of the land use associated with domestic food supply across a range of potential future scenarios. Large shifts in UK land use patterns could potentially lead to displacement of agricultural production to other countries, resulting in negative environmental consequences, which could partly negate the UK’s efforts to decarbonise the food system. The significance of GHG emissions embodied in food imports highlights a flaw in the accounting system of net-zero strategies, which consider only territorial emissions. Therefore, it is critical that we explore the full global impact of domestic land use policies.

## Supporting information

S1 File Supplementary methods and results. (PDF)
