# Supplementary material for: Global land footprint of UK food and feed imports under future socioeconomic scenarios
Source: PLoS One. 2026 Jun 30;21(6):e0352499. doi: 10.1371/journal.pone.0352499 (PMC13318043; doi:10.1371/journal.pone.0352499)
Supplement: S1 File — (PDF) [file pone.0352499.s001.pdf]

Supplementary Information for “Global land footprint of UK food and feed imports under future socioeconomic scenarios”

1 Supplementary Methods

1.1 Yield simulation and calibration

Crop yields were simulated by LPJ-GUESS version 4.1 as described in Alexander et al. (1) but using bias-adjusted ISIMIP-3b, CMIP6-based atmospheric climate forcings for the MRI-ESM2-0 model (2,3). Simulated yields were calibrated on a country level to observed yields from FAOSTAT (4) for 1995-2005, using historical nitrogen fertiliser use, irrigation, and crop distribution (see Alexander et al. (2018) for more information). For each crop, we derived a calibration factor using a linear model:

$$y_{obs,i,j,k} = \beta_i \cdot y_{sim,i,j,k} + \delta_i \cdot y_{sim,i,j,k} \cdot \ln(g_{j,k}) + \epsilon_{i,j,k}, \quad (1)$$

where $y_{obs,i,j,k}$ is the observed wet-matter crop yield, $y_{sim,i,j,k}$ is the simulated dry-matter crop yield, β_i and δ_i are estimated coefficients, $g_{j,k}$ is the GDP per capita (nominal), $\epsilon_{i,j,k}$ is the error term, i is the crop, j is the country, and k is the year. Observations were weighted by reported crop area. We included an interaction between GDP per capita and simulated yields to control for agricultural inputs other than nitrogen fertiliser use and irrigation, such as mechanisation and pesticide use. These other factors are simulated explicitly in PLUM (as management intensity) but not in LPJ-GUESS. We control for these factors so that the resulting calibration factors represent the maximum potential yield.

To calculate the yield calibration factors, we fixed GDP per capita (g) at the sample 90th percentile (representing a high level of agricultural intensification) such that the calibration factor for each crop i is:

$$calib\_factor_i = \beta_i + \delta_i ln(g). \quad (2)$$

The interaction term δ for *Miscanthus* was not significant and therefore the calibration was fitted using a simplified model without the interaction. Fig S1 shows the results of the calibration for each crop type.

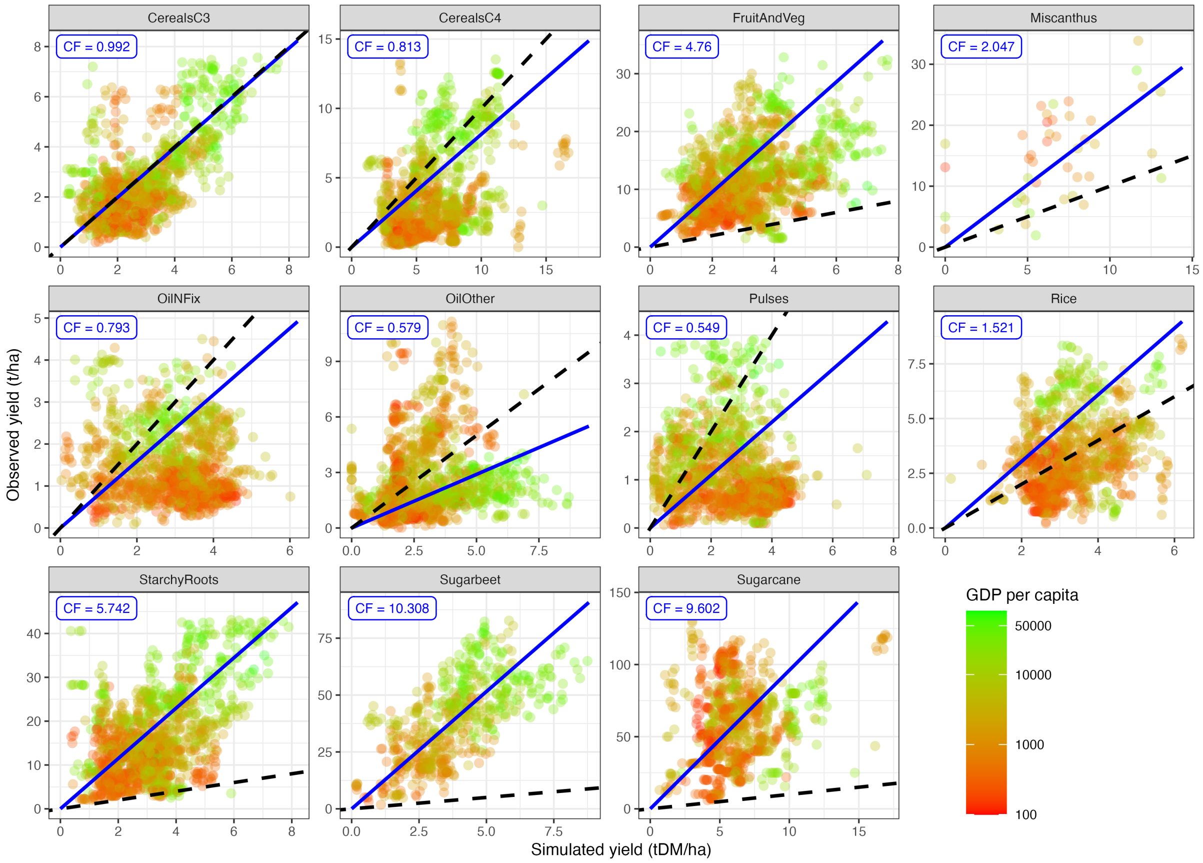

Fig S1 – Scatter plots illustrating the calibration of simulated dry-matter crop yields to observed wet-matter yields. Each point represents the average crop yield in one country for a given year between 1995 and 2005. Points are shaded by GDP per capita to illustrate the income dependence of yields. Crop calibration factors (CF) are shown in the top left corner of each panel. Solid blue lines show *y = calib_factor · x* and dashed black lines show *y = x* for comparison.

1.2 Food demand system

Food demand in PLUM is modelled endogenously by a Modified An Implicitly Directly Additive Demand System (MAIDADS) (5,6). MAIDADS predicts subsistence and discretionary per capita food and non-food demand using prices, average country income and fitted parameters which control the consumption bundle at each income level. We fitted the model following methods from Gouel and Guimbard (5). We used 2017 country-level, apparent food consumption (Food Balance Sheets) and producer prices as a proxy for

consumer prices from FAOSTAT (4). Prices were averaged over a 3-year period (2016-2018) to reduce interannual volatility. For each food commodity, missing prices were replaced with mean prices, first if available from prices from other years for the same country, then from other countries within the same year, and finally from all years and countries. For each country, prices were calculated as a weighted combination of domestic prices and global import prices, weighted by import and production amounts. Global import prices were calculated as a weighted average of producer prices of all exporters, weighted by export amount.

Reported GDP per capita (2017 $ PPP) and price level indices (PLI) were taken from the International Comparison Program 2017 (ICP) (7). We used the ratio of the non-food PLI to the food PLI as a proxy for non-food prices. This accounts for observed higher non-food prices relative to food prices in high income countries and allows us to model future non-food prices using an empirically derived relationship:

$$p_t = \frac{a + b \cdot \exp(x_t)}{1 + \exp(x_t)}; \qquad (3)$$

$$x_t = c \cdot (\ln(m_t) - d), \qquad (4)$$

where p_t is the non-food price proxy at time t, m_t is the GDP per capita (2017 $ PPP), and a, b, c, d are fitted parameters. Table S1 shows the fitted MAIDADS parameter values and Fig S2 shows the fitted consumption curves. In MAIDADS, parameters α and β control discretionary consumption, and δ and τ control subsistence consumption.

Commodity Group	δ	τ	α	β
Cereals & Starchy Roots	8.09E-01	5.46E-01	1.00E-06	0.00E+00
Sugar	4.88E-02	1.84E-01	9.68E-05	0.00E+00
Fruit & Veg	3.47E-02	1.51E-01	6.32E-03	0.00E+00
Pulses	5.49E-02	3.86E-03	1.41E-04	0.00E+00
Oil crops	1.09E-01	2.07E-01	3.30E-04	0.00E+00
Ruminants	1.15E-02	9.97E-02	1.54E-02	0.00E+00
Monogastrics	0.00E+00	7.75E-02	9.94E-03	0.00E+00
Non food	0.00E+00	0.00E+00	9.68E-01	1.00E+00
Other parameters: ω = 1.36; κ = 0.50.				

Table S1 – Fitted MAIDADS model parameters.

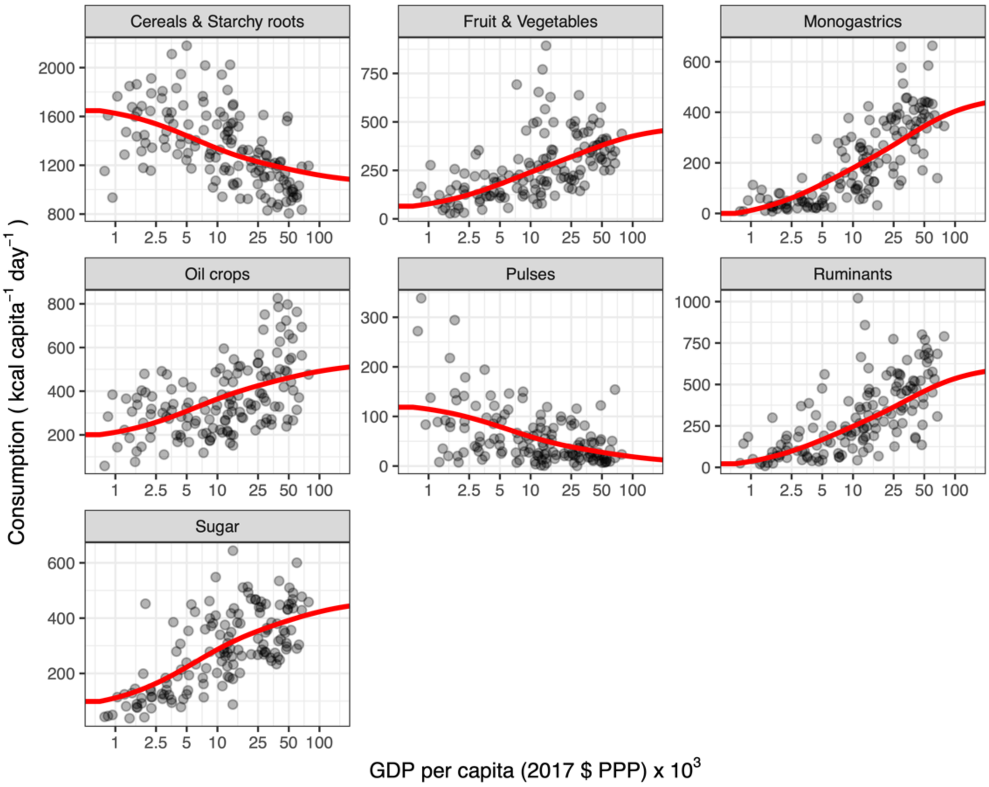

Fig S2 – Fitted demand curves predicted by MAIDADS (red lines) by food group and observed consumption (grey points).

Within PLUM, MAIDADS predicts per capita caloric demand on an annual timestep for seven food groups: cereals and starchy roots, oil crops, pulses, sugar, fruit and vegetables, ruminants, and monogastrics. Consumer food prices are computed by PLUM by projecting baseline (2017) consumer prices (proxied by producer prices; see above) based on relative changes in producer prices simulated in PLUM. Fig S3 illustrates the simulated food demand assuming constant 2017 prices. Per capita caloric demand is converted to mass demand using conversion factors calculated from FAOSTAT (mass of food supply divided by caloric content of food supply) and multiplied by the country's population to give total country demand. Demand for cereals and starchy roots is then disaggregated into demand for wheat and other C3 cereals, maize and other C4 cereals, rice, and starchy roots based on proportions from FAOSTAT in the baseline year (2020). Similarly, demand for oil crops is disaggregated into nitrogen-fixing oil crops (soya beans and groundnuts), and other oil crops. Demand predicted by MAIDADS is rebased to match observed consumption in 2020. We cap incomes at $50,000 to avoid extrapolating beyond the range of the data on which the model

was fitted. After this point, changes in demand are not sensitive to further increases in income but do still respond to changes in food prices.

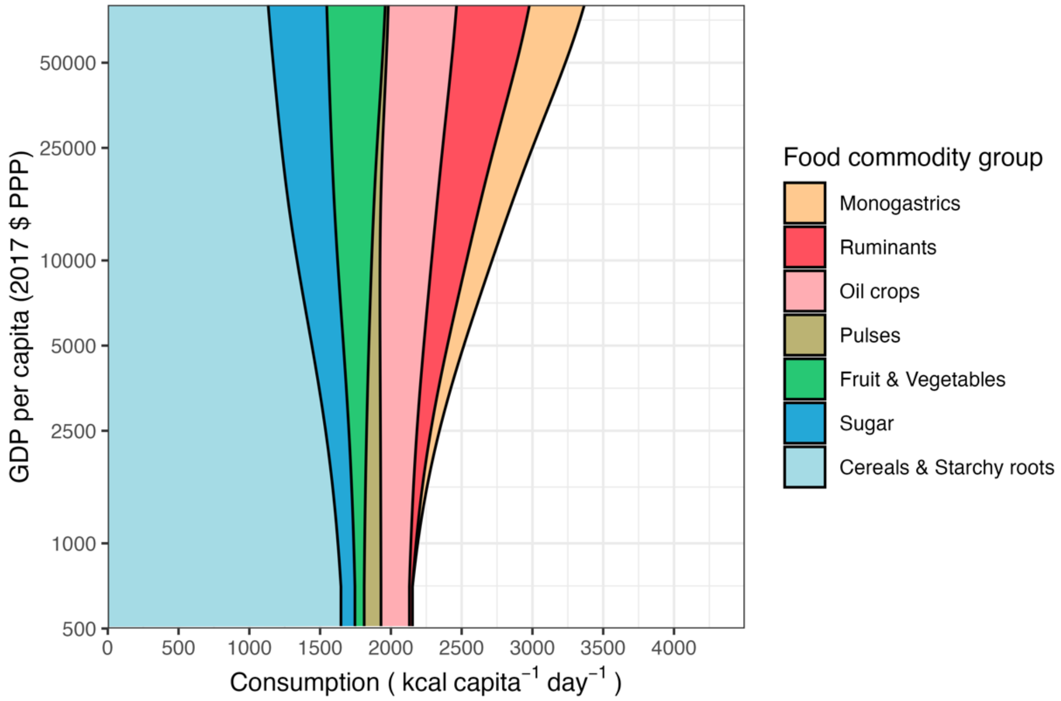

Fig S3 – Food demand predicted by MAIDADS for each food group with constant 2017 prices.

1.3 Dietary preferences

Changes in dietary preferences were modelled as a linear shift in subsistence (τ) and discretionary (α) parameters between 2020 and 2050. To calculate the target τ and α parameters, we first calculated an adjustment ratio for each food group using the EAT Lancet diet recommendation (8) and predicted high-income ($50,000) baseline food consumption from the fitted MAIDADS model (Table S2):

$$r_i = \frac{consumption_i^{EAT}}{consumption_i^{ref}}, \qquad (5)$$

Where r_i is the adjustment ratio for food group i, $consumption_i^{EAT}$ is the target consumption level from the EAT Lancet recommendation, and $consumption_i^{ref}$ is the reference consumption level from the fitted demand model at $50,000. Values from Willet et al. (2019) were adjusted to account for household food waste using a waste rate of 17% (9).

The target α and τ parameter values of each food group were then calculated from baseline parameter values using their corresponding adjustment ratios:

$$\alpha_i^* = r_i \cdot k \cdot \alpha + (1 - k) \cdot \alpha; \qquad (6)$$

$$\tau_i^* = r_i \cdot k \cdot \tau + (1 - k) \cdot \tau, \qquad (7)$$

where α_i^* and τ_i^* are the target parameters for food group i, α and τ are the reference parameters, and k is a scenario specific value (from 0 to 1) that determines the degree of change in dietary preferences. The following values of k were used in this study: 1 for SSP1, 0 for SSP3, 0.5 for SSP4, and 0 for SSP5. Given the constraint that $\sum_i \alpha_i = 1$ where i is the commodity group, we assume that the $\alpha_{nonfood} = 1 - \sum_{i \neq nonfood} \alpha_i$. During a model run, τ and α values were linearly interpolated between baseline values and target values over a period of 30 years between 2020 and 2050, after which point the parameters remained fixed at their target value.

Commodity Group	EAT Lancet (kcal/day)	Reference (kcal/day)	r_i
Cereals & Starchy Roots	995	1163	0.86
Fruit & Vegetables	413	398	1.04
Monogastrics	112	348	0.32
Oil crops	782	433	1.81
Pulses	201	29	7.01
Ruminants	239	461	0.52
Sugar	140	395	0.36

Table S2 – EAT Lancet (8) target diet, reference diet, and adjustment ratios (r_i).

When PLUM is initialised for the baseline year, an offset difference is calculated between predicted food demand and observed demand for each commodity. This offset is then added to the predicted demand during each timestep which rebases the predictions to match observed data in the baseline year. In scenarios where dietary preferences change over time, this offset is reduced linearly so that food demand converges towards that purely predicted by MAIDADS. The degree of convergence is determined by the scenario and is proportional to the degree of change in dietary preferences such that for each commodity:

$$\Delta_t = \Delta_{t_{start}} \cdot x_t, \tag{8}$$

$$x_t = \begin{cases} 1 - a \cdot \frac{t - t_{start}}{t_{end} - t_{start}}, & t_{start} \leq t \leq t_{end} \\ 1 - a, & t > t_{end} \\ 1, & t < t_{start} \end{cases} \tag{9}$$

Where Δ_t is the offset at time t, t_{start} and t_{end} are the start and end times of the convergence, and a is a scenario specific parameter, calculated as $0.7 \cdot k$ (= 0.7 for SSP1, 0 for SSP3, 0.35 for SSP4, and 0 for SSP5). The parameterisation of a was chosen so that there is a strong degree of convergence in scenarios with large shifts in dietary preferences (such as SSP1), but some country-specific dietary preferences are retained.

1.4 Socioeconomic factors

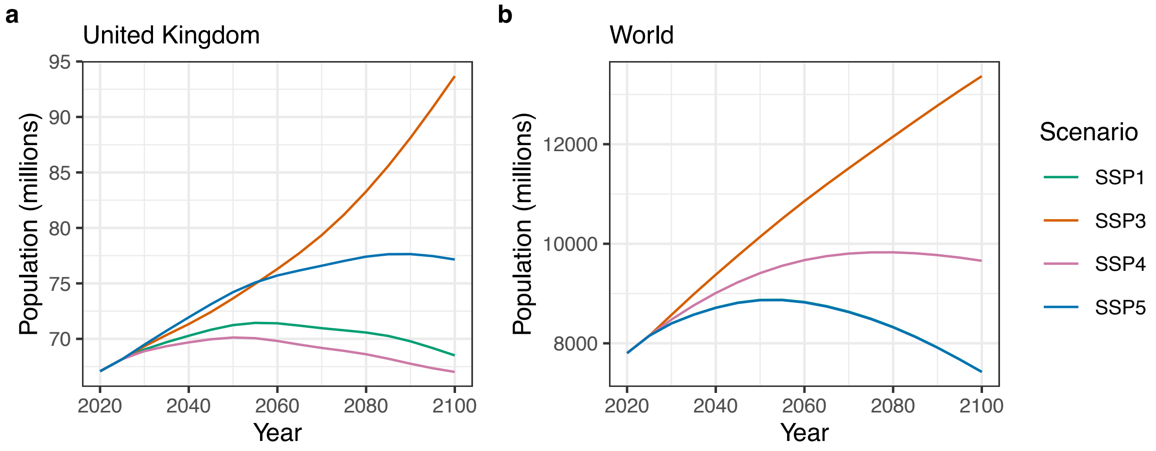

Fig S4 – Projected population for a) United Kingdom and b) World. Data from Koch & Leimbach, 2023 (10).

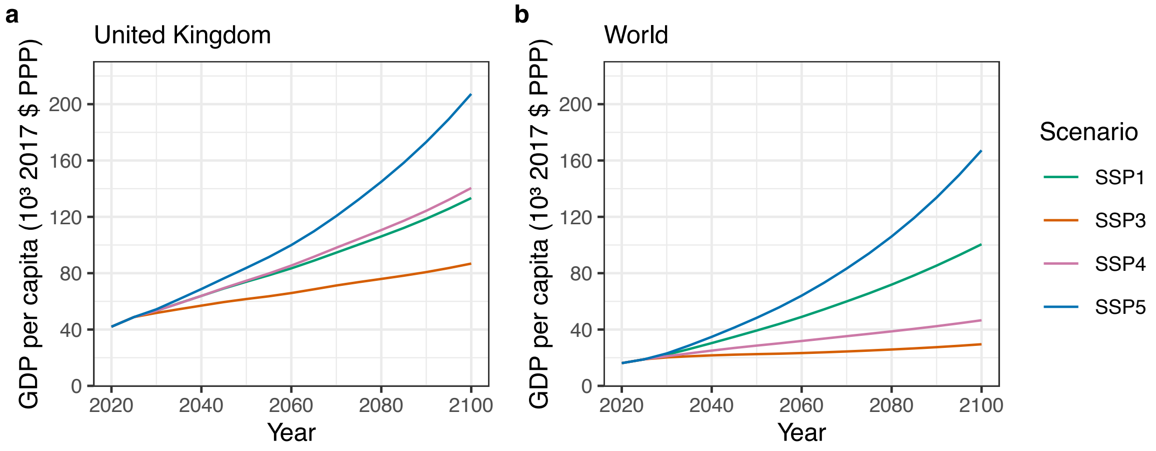

Fig S5 – Projected GDP per capita for a) United Kingdom and b) World. Data from Koch & Leimbach, 2023 (10).

1.5 Parameterisation

SSP	Parameter	Dist.	Min.	Max	P_5	Mode	P_{95}
SSP1	AGRI_LAND_EXPANSION_COST_FACTOR	beta	0	2	1.2	1.4	1.8
SSP3	AGRI_LAND_EXPANSION_COST_FACTOR	beta	0	2	0.3	0.5	0.8
SSP4	AGRI_LAND_EXPANSION_COST_FACTOR	beta	0	2	0.5	0.8	1
SSP5	AGRI_LAND_EXPANSION_COST_FACTOR	beta	0	2	0.5	0.8	1.2
SSP1	TRADE_ADJUSTMENT_COST_RATE	beta	0.003	0.015	0.004	0.005	0.006
SSP3	TRADE_ADJUSTMENT_COST_RATE	beta	0.003	0.015	0.007	0.009	0.011
SSP4	TRADE_ADJUSTMENT_COST_RATE	beta	0.003	0.015	0.006	0.007	0.009
SSP5	TRADE_ADJUSTMENT_COST_RATE	beta	0.003	0.015	0.004	0.005	0.006
SSP1	BIOENERGY_DEMAND_SHIFT	unif	0.9	1.1			
SSP3	BIOENERGY_DEMAND_SHIFT	unif	0.9	1.1			
SSP4	BIOENERGY_DEMAND_SHIFT	unif	0.9	1.1			
SSP5	BIOENERGY_DEMAND_SHIFT	unif	0.9	1.1			
SSP1	FERTILISER_COST_PER_T	beta	0.152	0.952	0.533	0.723	0.838
SSP3	FERTILISER_COST_PER_T	beta	0.152	0.952	0.228	0.419	0.647
SSP4	FERTILISER_COST_PER_T	beta	0.152	0.952	0.343	0.647	0.838
SSP5	FERTILISER_COST_PER_T	beta	0.152	0.952	0.228	0.343	0.533
SSP1	INITIAL_PRICE_SHIFT	unif	0.8	1.2			
SSP3	INITIAL_PRICE_SHIFT	unif	0.8	1.2			
SSP4	INITIAL_PRICE_SHIFT	unif	0.8	1.2			
SSP5	INITIAL_PRICE_SHIFT	unif	0.8	1.2			
SSP1	IRRIG_COST_SCALE_FACTOR	beta	0.00005	0.00065	0.00035	0.00045	0.00055
SSP3	IRRIG_COST_SCALE_FACTOR	beta	0.00005	0.00065	0.0001	0.0002	0.00025
SSP4	IRRIG_COST_SCALE_FACTOR	beta	0.00005	0.00065	0.00015	0.00035	0.00055
SSP5	IRRIG_COST_SCALE_FACTOR	beta	0.00005	0.00065	0.0001	0.0002	0.00035
SSP1	IRRIGATION_EFFICIENCY	beta	0.4	0.6	0.52	0.55	0.58
SSP3	IRRIGATION_EFFICIENCY	beta	0.4	0.6	0.42	0.45	0.48
SSP4	IRRIGATION_EFFICIENCY	beta	0.4	0.6	0.45	0.5	0.55
SSP5	IRRIGATION_EFFICIENCY	beta	0.4	0.6	0.5	0.52	0.55
SSP1	LAND_CONVERSION_COST_FACTOR	beta	0.25	1.75	1.125	1.375	1.5
SSP3	LAND_CONVERSION_COST_FACTOR	beta	0.25	1.75	0.75	1	1.25
SSP4	LAND_CONVERSION_COST_FACTOR	beta	0.25	1.75	1	1.125	1.375
SSP5	LAND_CONVERSION_COST_FACTOR	beta	0.25	1.75	0.5	0.75	0.875
SSP1	MARKET_LAMBDA	unif	0.8	1.2			

SSP3	MARKET_LAMBDA	unif	0.8	1.2			
SSP4	MARKET_LAMBDA	unif	0.8	1.2			
SSP5	MARKET_LAMBDA	unif	0.8	1.2			
SSP1	MEAT_EFFICIENCY	beta	0.95	1.05	1.01	1.02	1.04
SSP3	MEAT_EFFICIENCY	beta	0.95	1.05	0.98	1	1.02
SSP4	MEAT_EFFICIENCY	beta	0.95	1.05	0.98	1	1.02
SSP5	MEAT_EFFICIENCY	beta	0.95	1.05	1	1.01	1.02
SSP1	MIN_NATURAL_RATE	beta	0	0.3	0.14	0.2	0.25
SSP3	MIN_NATURAL_RATE	beta	0	0.3	0.02	0.04	0.07
SSP4	MIN_NATURAL_RATE	beta	0	0.3	0.04	0.06	0.1
SSP5	MIN_NATURAL_RATE	beta	0	0.3	0.02	0.04	0.06
SSP1	OTHER_INTENSITY_COST	beta	0.54	1.06	0.8	0.94	1
SSP3	OTHER_INTENSITY_COST	beta	0.54	1.06	0.6	0.74	0.86
SSP4	OTHER_INTENSITY_COST	beta	0.54	1.06	0.66	0.86	1
SSP5	OTHER_INTENSITY_COST	beta	0.54	1.06	0.6	0.66	0.8
SSP1	PASTURE_HARVEST_FRACTION	unif	0.4	0.6			
SSP3	PASTURE_HARVEST_FRACTION	unif	0.4	0.6			
SSP4	PASTURE_HARVEST_FRACTION	unif	0.4	0.6			
SSP5	PASTURE_HARVEST_FRACTION	unif	0.4	0.6			
SSP1	SSP_GDP_PC_FACTOR	unif	0.9	1.1			
SSP3	SSP_GDP_PC_FACTOR	unif	0.9	1.1			
SSP4	SSP_GDP_PC_FACTOR	unif	0.9	1.1			
SSP5	SSP_GDP_PC_FACTOR	unif	0.9	1.1			
SSP1	SSP_POPULATION_FACTOR	unif	0.9	1.1			
SSP3	SSP_POPULATION_FACTOR	unif	0.9	1.1			
SSP4	SSP_POPULATION_FACTOR	unif	0.9	1.1			
SSP5	SSP_POPULATION_FACTOR	unif	0.9	1.1			
SSP1	TECHNOLOGY_CHANGE_ANNUAL_RATE	beta	-0.004	0.008	0.003	0.005	0.007
SSP3	TECHNOLOGY_CHANGE_ANNUAL_RATE	beta	-0.004	0.008	-0.001	0	0.001
SSP4	TECHNOLOGY_CHANGE_ANNUAL_RATE	beta	-0.004	0.008	0	0.002	0.005
SSP5	TECHNOLOGY_CHANGE_ANNUAL_RATE	beta	-0.004	0.008	0.002	0.004	0.005
SSP1	TRADE_BARRIER_MULTIPLIER	beta	0	2	0.6	0.8	1
SSP3	TRADE_BARRIER_MULTIPLIER	beta	0	2	1.2	1.5	1.8
SSP4	TRADE_BARRIER_MULTIPLIER	beta	0	2	1	1.2	1.5
SSP5	TRADE_BARRIER_MULTIPLIER	beta	0	2	0.3	0.6	0.8
SSP1	TRANSPORT_COST	beta	0.01	0.09	0.05	0.065	0.08
SSP3	TRANSPORT_COST	beta	0.01	0.09	0.02	0.04	0.06
SSP4	TRANSPORT_COST	beta	0.01	0.09	0.04	0.06	0.08

SSP5	TRANSPORT_COST	beta	0.01	0.09	0.02	0.035	0.05

Table S3 – Model parameter distributions per SSP.

1.6 Estimation of import ratios

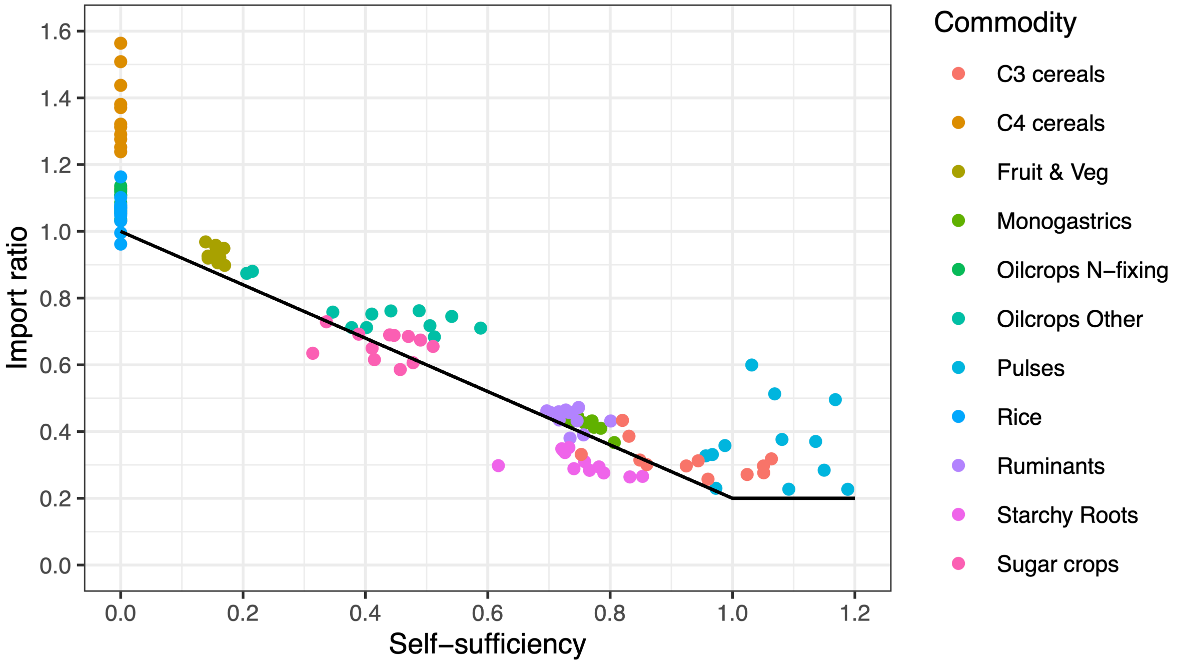

Fig S6 - UK import ratio against self-sufficiency between 2010 and 2021. Each point represents a single year and commodity. Solid black line shows the modelled relationship between self-sufficiency and the import ratio.

2 Supplementary results

Crop	Footprint (Mha)	Crop	Footprint (Mha)
Soya beans	1.201	Sugar cane	0.077
Wheat	1.014	Beans	0.074
Cocoa beans	0.781	Triticale	0.074
Maize (corn)	0.752	Oranges	0.068
Rapeseed	0.499	Rye	0.061
Coffee	0.377	Groundnuts	0.060
Sunflower seed	0.355	Oats	0.057
Barley	0.327	Sugar beet	0.056
Grapes	0.286	Apples	0.055
Rice	0.257	Coconuts	0.055
Olives	0.203	Chickpeas	0.054
Cashew nuts	0.133	Other crops	0.895

Table S4 – Individual contributions of crops to the total cropland footprint of UK food and feed imports (including feed embodied in animal products). Only crops with a footprint of at least 0.05 Mha are shown and the rest are aggregated as “Other crops”.

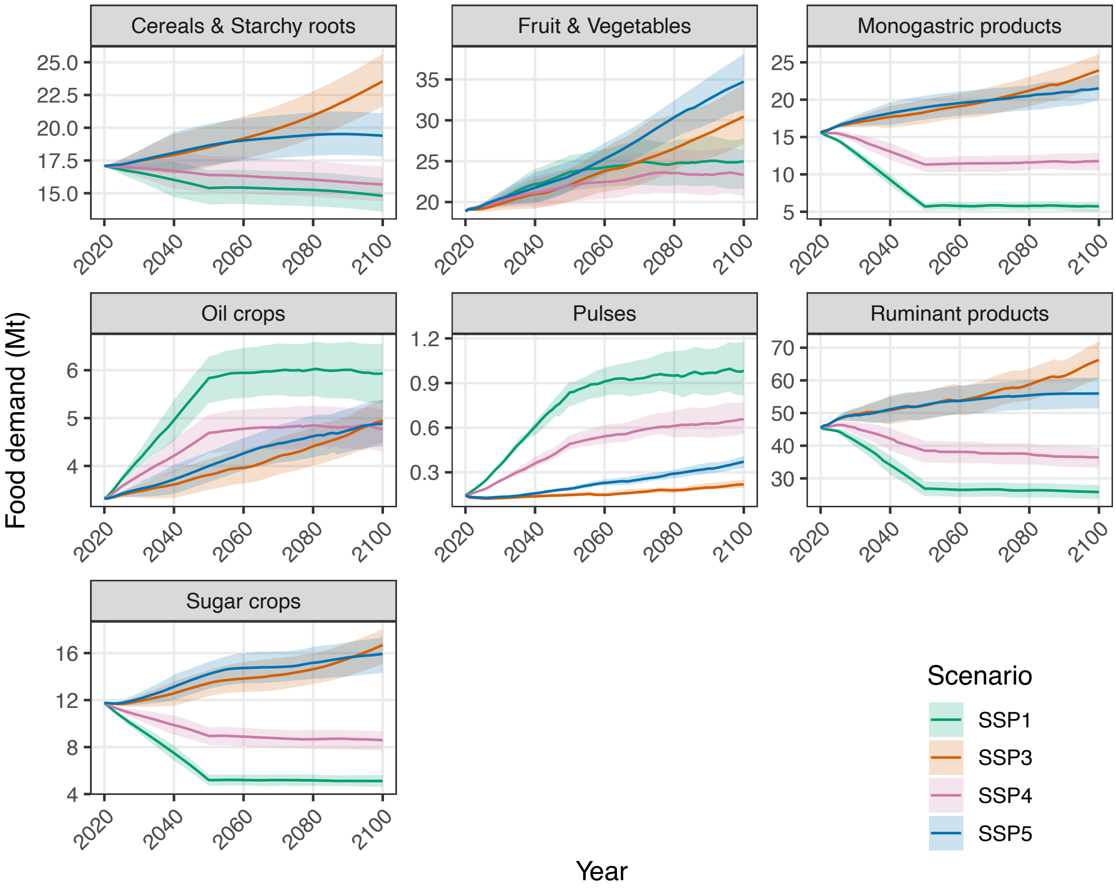

Fig S7 – Simulated total food demand in the UK by commodity. Monogastric and ruminant demand are reported in dry matter feed equivalent amounts. All other quantities are in wet matter amounts.

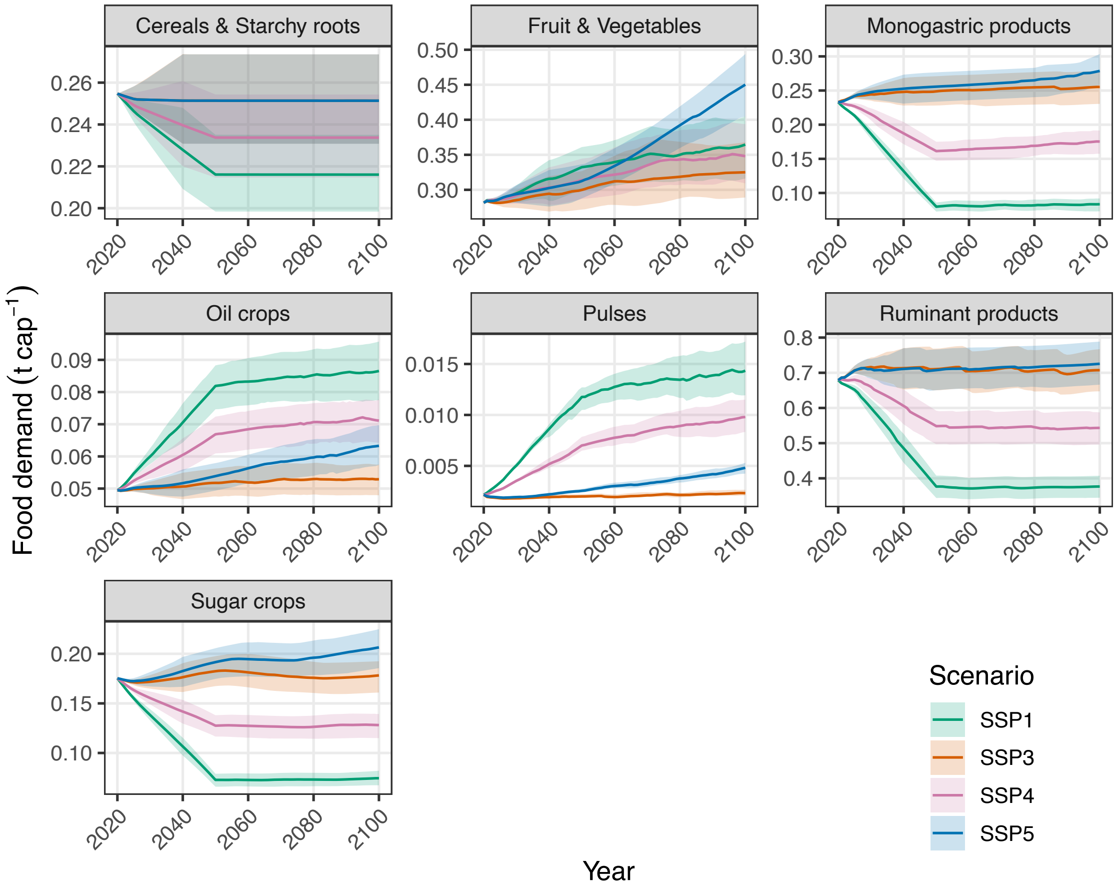

Fig S8 – Simulated per capita food demand in the UK by commodity. Monogastric and ruminant demand are reported in dry matter feed equivalent amounts. All other quantities are in wet matter amounts.

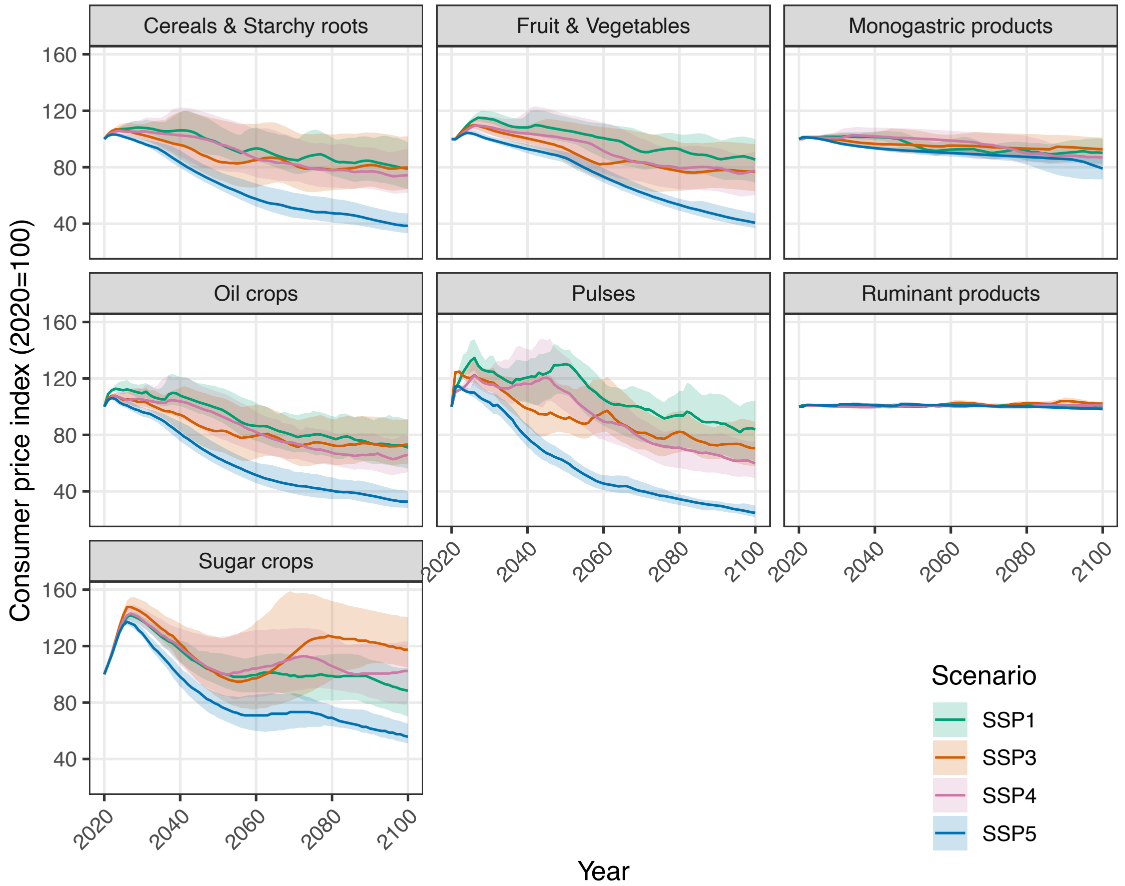

Fig S9 – Simulated consumer price index in the UK by commodity.

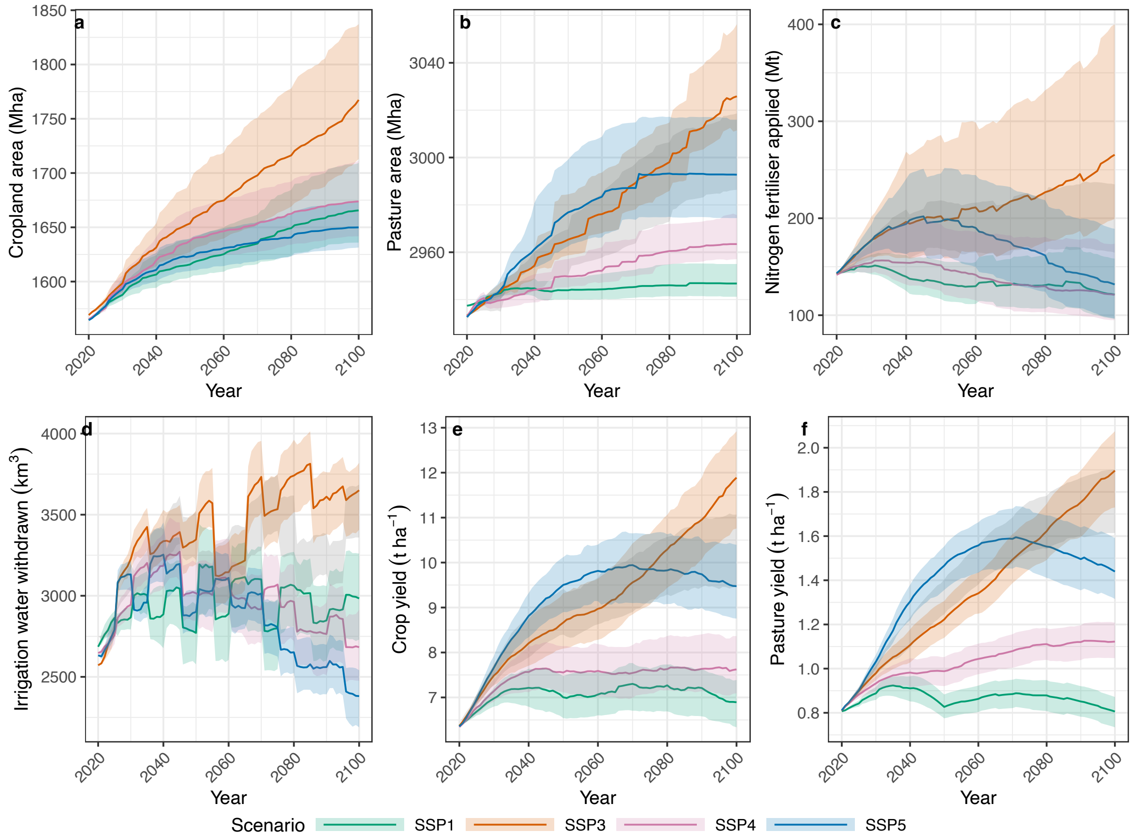

Fig S10 – Simulated global land use statistics from 2020 to 2100 including a) cropland area, b) pasture area, c) nitrogen fertiliser applied, d) irrigation water withdrawn, e) average crop yield, and f) average pasture yield.

2.1 Baseline comparisons

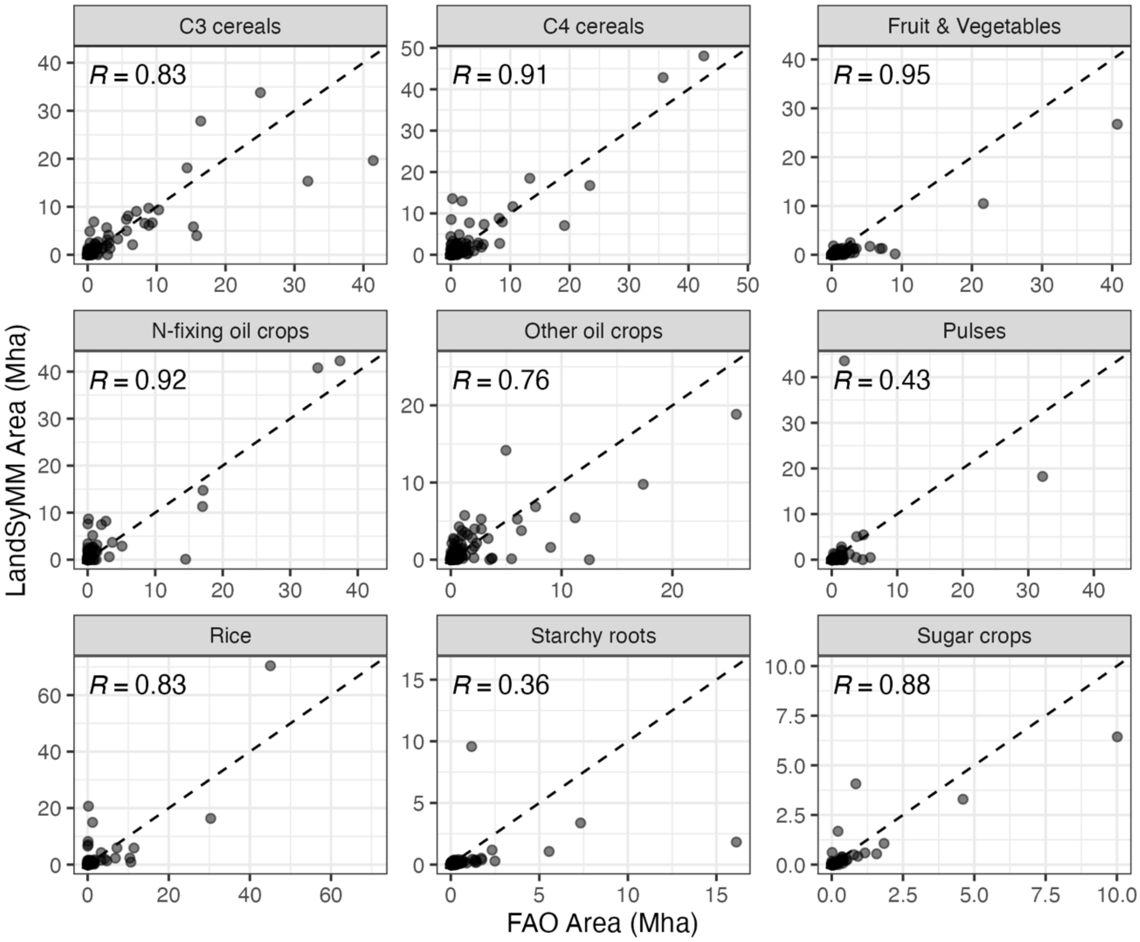

Fig S11 – Country-wise comparison of reported (FAO) and simulated (LandSyMM) crop areas in 2020. Pearson correlation coefficients are shown in the top left corner of each panel. Dashed lines show y=x.

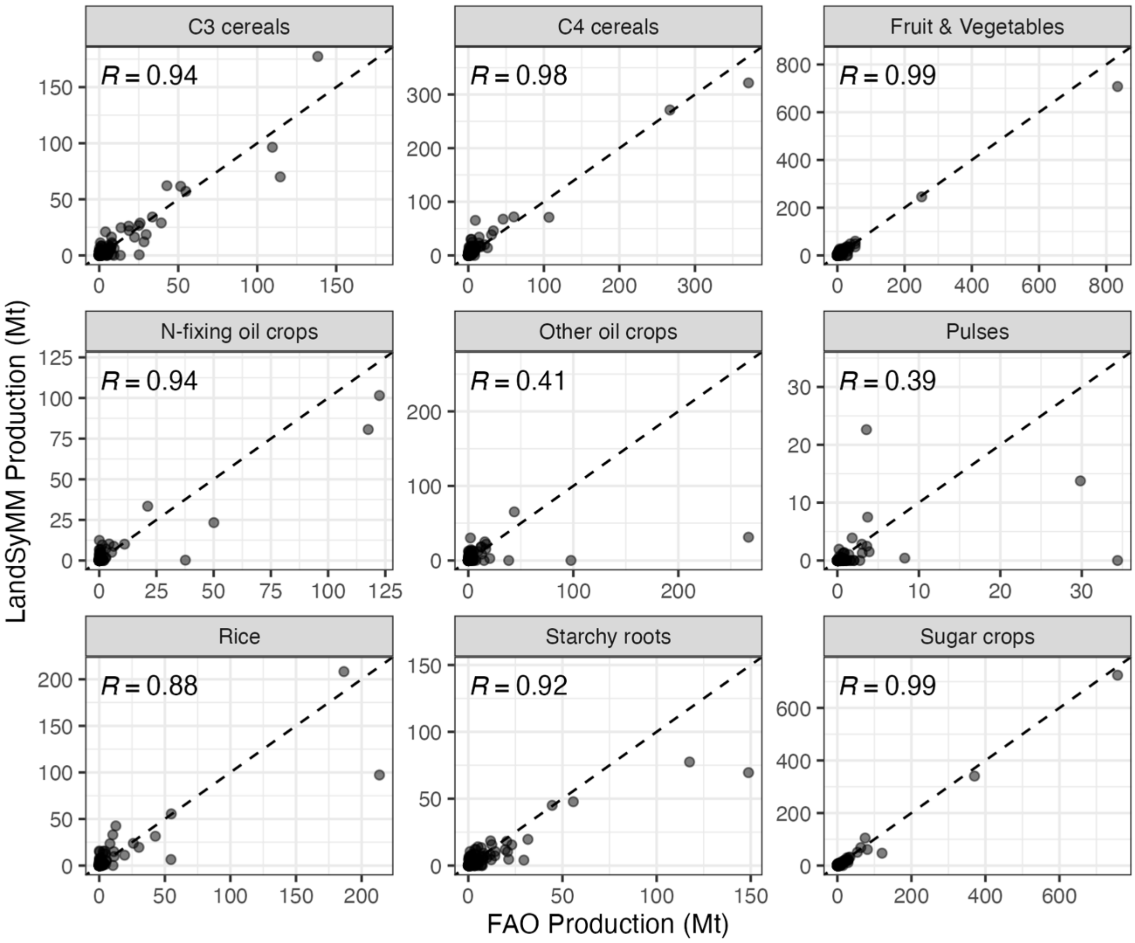

Fig S12 – Country-wise comparison of reported (FAO) and simulated (LandSyMM) crop production in 2020. Pearson correlation coefficients are shown in the top left corner of each panel. Dashed lines show y=x.

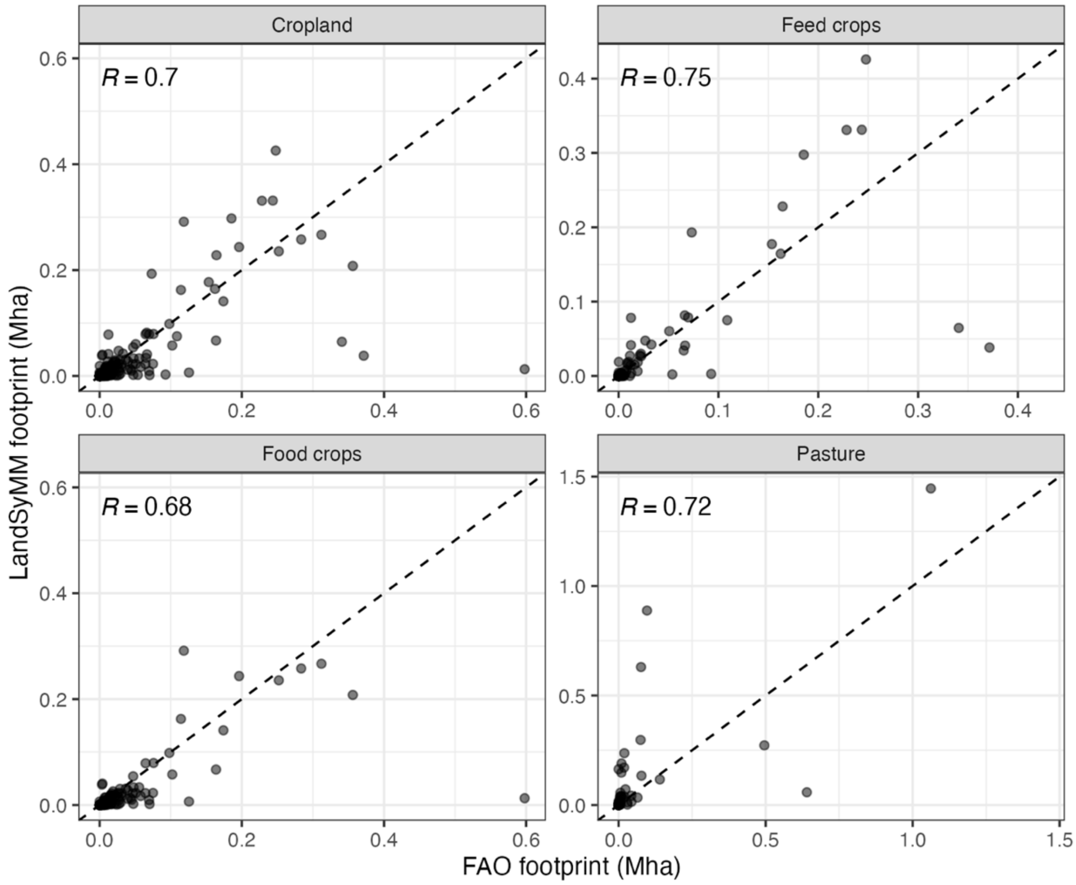

Fig S13 – Country-wise comparison of reported (FAO) and simulated (LandSyMM) land footprints of UK imports in 2020 for (clockwise) cropland, feed crops, pasture and food crops. Pearson correlation coefficients are shown in the top left corner of each panel. Dashed lines show y=x.

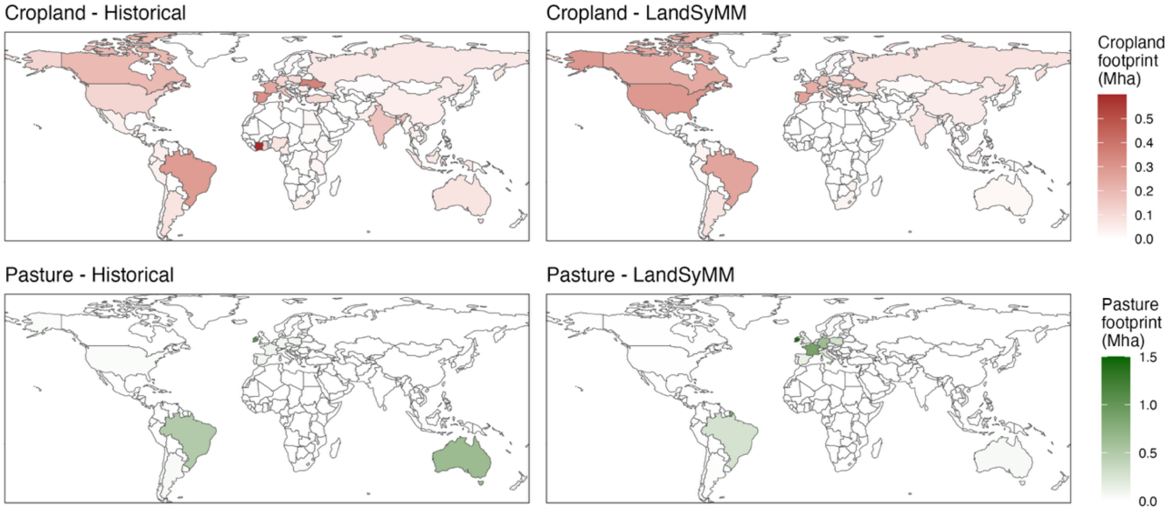

Fig S14 – Maps showing total land footprint of UK imports for cropland (top row) and pasture (bottom row) by country in 2020 based on reported FAO data (left column) and simulated LandSyMM data (right column). Made with Natural Earth (https://www.naturalearthdata.com).

3 References

1. Alexander P, Rabin S, Anthoni P, Henry R, Pugh TAM, Rounsevell MDA, et al. Adaptation of global land use and management intensity to changes in climate and atmospheric carbon dioxide. Global Change Biology. 2018;24(7):2791–809.

2. Lange S, Büchner M. ISIMIP3b bias-adjusted atmospheric climate input data [Internet]. ISIMIP Repository; 2021. Available from: https://doi.org/10.48364/ISIMIP.842396.1

3. Yukimoto S, Koshiro T, Kawai H, Oshima N, Yoshida K, Urakawa S, et al. MRI MRI-ESM2.0 model output prepared for CMIP6 [Internet]. Earth System Grid Federation; 2019 [cited 2023 Dec 5]. Available from: https://doi.org/10.22033/ESGF/CMIP6.621

4. FAO. FAOSTAT [Internet]. 2023 [cited 2023 Nov 4]. Available from: https://www.fao.org/faostat

5. Gouel C, Guimbard H. Nutrition Transition and the Structure of Global Food Demand. American Journal of Agricultural Economics. 2019;101(2):383–403.

6. Preckel PV, Cranfield JAL, Hertel TW. A modified, implicit, directly additive demand system. Applied Economics. 2010 Jan 1;42(2):143–55.

7. The World Bank. Purchasing Power Parities and the Size of World Economies: Results from the 2017 International Comparison Program [Internet]. The World Bank; 2020. Available from: https://elibrary.worldbank.org/doi/abs/10.1596/978-1-4648-1530-0

8. Willett W, Rockström J, Loken B, Springmann M, Lang T, Vermeulen S, et al. Food in the Anthropocene: the EAT–Lancet Commission on healthy diets from sustainable food systems. The Lancet. 2019 Feb;393(10170):447–92.

9. UNEP. UNEP Food Waste Index Report 2021 [Internet]. 2021 [cited 2023 Nov 3]. Available from: http://www.unep.org/resources/report/unep-food-waste-index-report-2021

10. Koch J, Leimbach M. SSP economic growth projections: Major changes of key drivers in integrated assessment modelling. Ecological Economics. 2023 Apr 1;206:107751.
