# Supplementary material for: Global land footprint of UK food and feed imports under future socioeconomic scenarios
Source: PLoS One. 2026 Jun 30;21(6):e0352499. doi: 10.1371/journal.pone.0352499 (PMC13318043; doi:10.1371/journal.pone.0352499)
Supplement: Attachment — Submitted filename: Response To Reviewers.docx [file pone.0352499.s003.docx]

**Reviewer 1**

| Reviewer’s comments | Authors’ response |
| --- | --- |
| Reviewer #1: This manuscript presents an in-depth analysis of the global land footprint associated with the UK’s food and feed imports, both historically (2010–2020) and under future scenarios up to 2100. Using a combination of historical FAOSTAT data and simulations from the Land System Modular Model (LandSyMM), the authors estimate the land use impacts of UK consumption across four socioeconomic scenarios. The integration of advanced land use modeling with scenario analysis provides valuable insights for both academic researchers and policymakers. However, there are still some contents that need to be carefully revised before final publication. | We thank the reviewer for their time and valuable feedback. |
| 1. The methodological section is comprehensive but would benefit from greater clarity and conciseness. While the integration of the LandSyMM framework, including LPJ-GUESS and PLUM, is described in detail, summarizing the overall workflow with a schematic or flowchart would make it more accessible and easier to follow. | We have included a schematic (Fig 1) to illustrate the coupling between PLUM and LPJ-GUESS as well as links to input data sources such as the FAO. |
| 2. The manuscript employs four SSP-RCP scenarios (SSP1-RCP2.6, SSP3-RCP7.0, SSP4-RCP6.0, and SSP5-RCP8.5) to explore the future global land footprint of UK food and feed imports. However, SSP2, which is widely used in multi-scenario analyses as a reference or “middle-of-the-road” scenario, is notably absent. Why was SSP2 excluded from the analysis, given its common use in scenario-based studies for comparative analysis? The manuscript should provide a clear rationale for excluding SSP2 and explain why the selected scenarios sufficiently capture the spectrum of possible future trajectories. | The four SSP-RCP scenarios presented in the study were chosen to a) produce a set of distinctive results and b) limit the number of scenarios to improve clarity and ease of interpretability. We believe that including SSP2 would not have produced any more relevant results, given that the socioeconomic variables of this scenario are broadly intermediate between the other scenarios. SSP2 is generally overrepresented in the literature, with many studies focusing solely on this scenario. By focusing on just four SSP-RCP combinations with clear socioeconomic distinctions (Table 1), a clearer narrative can be established. While SSP2 is frequently used as a reference scenario, its underlying socio-economic assumptions largely represent a continuation of historical trends and typically produce land-use, agricultural productivity, and trade outcomes that lie between the more divergent pathways represented by SSP1 and SSP5, depending on the associated concentration pathway. As the objective of this study is to explore the sensitivity of the UK’s global food and feed land footprint to structurally distinct socio-economic futures - rather than to provide an interpolative benchmark - we focus on a set of SSP-RCP combinations that span contrasting mechanisms relevant to land demand: sustainability-driven demand reduction (SSP1-RCP2.6), regional fragmentation and low productivity growth (SSP3-RCP7.0), inequality and externalization of land use (SSP4-RCP6.0), and fossil-fueled development with high consumption and intensification (SSP5-RCP8.5). Together, these scenarios capture a broad envelope of plausible future trajectories, within which SSP2-based outcomes would be expected to fall and which are unlikely to alter the qualitative conclusions of the analysis. This has now been clarified in the manuscript. |
| 3. The manuscript assumes that trade preferences remain constant throughout the 21st century, meaning the share of commodities imported from specific exporting countries does not change over time. While this simplifies the modeling process, it is a significant limitation that may not accurately reflect the dynamic nature of international trade influenced by geopolitical, economic, and environmental factors. For example, recent global events, such as the trade wars initiated by the Trump administration (e.g., increasing tariffs on key imports), demonstrate how trade preferences and patterns can shift rapidly due to policy decisions. Similarly, the ongoing emphasis on reducing reliance on specific regions for critical goods (e.g., reducing dependency on China for supply chains) or addressing environmental concerns (e.g., the EU’s deforestation-free trade regulations) suggests that trade preferences are likely to evolve significantly in the future. The manuscript should explicitly discuss the limitations of assuming constant trade preferences and how this assumption might affect the reliability of the results. For instance, how would shifts in trade relationships (e.g., increased tariffs, sanctions, or new trade agreements) impact the projected land footprint? | We agree that the assumption of fixed bilateral trade relations is a limitation of this study. Future work could improve on this aspect by making explicit predictions about changing bilaterial trade patterns, for example, by incorporating a gravity trade model. While changes in trade preferences due to macroeconomic factors may be in some way predictable, those relating to political factors (as described by the reviewer) are not foreseeable. Additionally, recent events such as the changes to US import tariffs are beyond the scope of this study given that simulations commenced before these were announced (both in terms of the baseline year of the model, 2020, and when the results were produced). Finally, the long temporal scope of the simulations (2020-2100) means that that incorporating potential changes to global trade patterns due to recent factors would also require predicting how long those factors may be relevant. These assumptions would be difficult to justify given the rapidly changing geopolitical situation. Therefore, by basing simulations on constant trade preferences, we avoid the need to predict changing geopolitical factors while incorporating some information about likely future trade patterns (which are likely to be similar to present day patterns). Parts of this response have been incorporated into the manuscript. |
| 4. Figure 1 shows that in SSP1 and SSP4, which represent shifts towards healthier and more sustainable diets (e.g., increased consumption of fruits and vegetables and reduced consumption of animal products), the projected imports of vegetables and fruits from 2080 to 2100 are smaller than those in SSP3, which assumes no dietary shift. This trend is counterintuitive, as healthier diets are expected to increase fruit and vegetable consumption. Could this result be explained by differences in population growth across the scenarios? For example, SSP3 assumes higher population growth, which might offset the lower demand per capita. However, the manuscript does not explicitly present the population trajectories or other key socioeconomic drivers for each scenario, making it difficult to interpret the underlying causes of this trend. In addition, the results for C4 cereals, other oil crops, N-fixing oil crops, rice, and pulses show remarkably small differences across the four scenarios. This contrasts with the significant variation observed for other food groups, such as fruits, vegetables, and animal products. The manuscript does not explain why these crop categories are relatively unaffected by the dietary shifts and socioeconomic changes modeled in the scenarios. Is this due to inherent assumptions in the LandSyMM model (e.g., limited scope for change in demand or production for these crops), or are these crops less sensitive to population and dietary shifts in the UK? | The increase in fruit and vegetable consumption in SSP3 is primarily due to high population growth. In LandSyMM, food demand is determined by a combination of exogenously specified dietary preferences, incomes and population, and endogenously calculated prices. Therefore, while there are large differences in dietary preferences, for example between SSP1 and SSP3, these do not necessarily lead to the same differences in aggregate demand due to changes in incomes and population which can mitigate or amplify changes in dietary preferences. Additionally in the case of fruit and vegetables, applying the EAT Lancet recommendation only increases the demand preference by 4% (Table S2 in S1 File). Therefore, differences in fruit and veg demand between scenarios are largely driven by other factors such as income and prices.  For completeness, we have now included additional figures in the Supplementary Information to illustrate changes in socioeconomic factors and diet in the UK. Supplementary Figures 4 and 5 show projected population and GDP per capita used as inputs to the model. Figures S7 and S8 in S1 File show to the total and per capita food demand for the United Kingdom. These show clear reductions in demand for monogastric and ruminant products, and sugar in SSP1 and SSP4. We note that while transition to an EAT Lancet diet results in an 81% caloric increase in oil crops consumption (Table S2 in S1 File) this results in a smaller mass increase (in tonnes) due to the high calorie density of oil crop products. Additionally, we see an increase in fruit and vegetable demand in SSP5 compared to other scenarios due to a large reduction in fruit and veg prices driven by increased yields from CO_2_ fertilisation and model assumptions about land management costs.  A summary of this discussion and reference to the supplementary figures has been added to the methods section.  Finally, we have rescaled each panel in Fig 1 to more clearly show the simulated changes in imports for each commodity. Previously, certain crops (e.g. oil crops) appeared to show little difference between scenarios. This was purely due how these were plotted with a fixed scale across all crops. It is clear now that dietary change and socioeconomic factors are having a significant impact on import levels, for example leading to a 60% increase in N-fixing oil crop imports in SSP1. |
| 5. The manuscript provides detailed projections of the UK’s land footprint under different SSP-RCP scenarios, but some results lack clear and convincing explanations, which raises concerns about the robustness and interpretability of the findings. For instance, regarding lines 452–458, the results indicate that despite higher population growth and increased demand for animal products in SSP3, the land footprint does not increase as one might intuitively expect. Instead, the SSP3 land footprint remains comparable to or even slightly lower than other scenarios. Although some justification is offered in lines 460–464—namely, that higher agricultural intensity mitigates land footprint growth—this explanation is not fully convincing or substantiated within the current manuscript. Given the centrality of this counterintuitive result, I believe the authors should provide a much more thorough and transparent analysis. Specifically, the manuscript should: 1) clearly describe the mechanisms in the model that allow increased demand to be offset by agricultural intensification; 2) present supporting data or sensitivity analyses demonstrating how changes in yields, management practices, or trade patterns in SSP3 lead to a stable or reduced land footprint; 3) compare these findings more directly to previous literature and discuss to what extent they are consistent or divergent. | Thank you for highlighting this gap in our analysis. We have now included Fig S10 to show the evolution of global land use in our simulations and to support our explanations of how land footprints differ between the scenarios. The central tenet of this study is that land footprints are a factor of both consumption level and agricultural intensity. Therefore, both factors must be considered to understand how the UK’s import land footprint evolves under each scenario. In both SSP3 and SSP5, greater imports are balanced by rapid increases in global agricultural intensity. This increase in intensity occurs for contrasting reasons which drive food demand: in SSP3 due to rapid population growth and in SSP5 due to rapid income growth and scenario parameterisation which favours intensification. The patterns of agricultural intensification and expansion across the scenarios have a significant impact of the UK’s import land footprint and therefore trends in the simulated footprint may not entirely mirror the trend in import levels.  This trade-off between agricultural intensification and expansion is simulated explicitly in LandSyMM. When demand increases, the model increases domestic supply through a combination of expansion, intensification, and imports determined through least cost optimisation. Subsequently, changes in demand-supply balances lead to adjustment in global prices which then impact demand of price-elastic commodities. Validation of this process is beyond the scope of this study but has been explored previously in detail by (Alexander et al., 2018) who show that model results are largely consistent with historical trends in global land use. |

**Reviewer 2**

| Reviewer’s comments | Authors’ response |
| --- | --- |
| Reviewer #2: This manuscript, entitled ‘Global land footprint of UK food and feed imports under future socioeconomic scenario’ (PONE-D-25-11636), represents the first systematic assessment of the global land footprint of the UK's food and feed imports, combining historical data with future scenario simulations. It demonstrates clear practical significance and methodological innovation. The research design is well-structured, employing advanced methodologies with transparent data sources. There are some obvious problems, and I would suggest that they be significantly revised before further consideration. | We thank the reviewer for their time and valuable feedback. |
| 1. It is unclear whether the global-scale land system model LandSyMM possesses sufficient resolution and accuracy at the national level (e.g., the United Kingdom). It is recommended to supplement the model with validation of its applicability at the national scale or supporting literature. | Historical validation of the model has been explored by (Alexander et al., 2023). Food demand is projected on a country level using baseline data for 2020 from the FAO and a price and income elastic demand model (MAIDADS) fitted on historical data (Gouel & Guimbard, 2019)). The model takes input data from the dynamic global vegetation model LPJ-GUESS which has been compared and validated against observations and other models (Ma et al., 2022; Müller et al., 2021; Olin et al., 2015). Land use, including fertiliser application, irrigation, other management, crop areas and crop yields are simulated explicitly on a 0.5-degree grid (approx. 55km2 at the equation). Net trade is simulated for each country with a single global international market. In summary, the model operates on a range of levels from global to country to grid-cell which makes it appropriate for exploring the food and land system at the national level as shown in this study. Similar work has been previously published, for example, exploring the impacts of the war in Ukraine on global land use (Alexander et al. 2023).  Additionally, we highlight that this study focuses not on land use in the UK per se, but rather on the UK’s impact on global land use for which a global-scale model such as LandSyMM is required. While the model is not specifically tailored for the UK, we don’t believe this is a limitation given that our analysis focuses primarily on global patterns. |
| 2. It is necessary to supplement the technology roadmap and detailed explanations. This helps readers from other fields quickly grasp the research structure and methodology. | We have added a schematic (Fig 1) to illustrate the methodology. |
| 3. It is recommended to insert spatial distribution maps of land footprints for key years or scenarios into the main text to enhance visual representation and policy interpretation capabilities. | This information was presented in supplementary figures which show the change in the land footprint for period 2020-2050 and 2020-2100. We have moved these to the main manuscript replacing the previous figure showing just SSP1. See Fig 7 and 8. |
| 4. The model employs historical data from 2010 to 2020 and comprehensive scenario assumptions to project long-term land footprint trends from 2020 to 2100. However, a significant concern arises: can the limited historical data and numerous assumptions sufficiently support the reliability of such extended-term projections? The authors must ensure enhanced the article's findings. | The results presented here should be interpreted as a “what if” analysis rather than forming any predictive claim about future land use. We focus on four distinct scenarios to explore how the UK global footprint could evolve under change socioeconomic and climatic factors. The factors driving the results presented in this study, including population growth, economic growth, and changes in dietary preferences cannot be predicted with certainty and therefore we cannot make any claims as to the reliability of these assumptions. Instead, the results should be interpreted as a range of possible outcomes that are plausible given the assumptions.  Nevertheless, the simulated land footprints align relatively well with the historical estimates (Fig 6), with the total land footprint of UK feed and food imports being comparable. This suggests the model is able to reproduce the expected present day patterns of the UK’s global land footprint and therefore make informed estimates of how these could evolve in the future. |
| 5. Although “stochastically sampled input parameters” are mentioned, the study does not specify which parameters were sampled, the sampling distribution used, or whether sensitivity analysis was performed. It is recommended to supplement the details of the uncertainty analysis to enhance the credibility of the results. | We have added additional information in the supplementary material about model parameterisation (Table S3) |
| 6. The author conducted historical research and projections on the food land footprint generated by British feed imports, but did not delve deeply into the resource and environmental impacts resulting from trade imports. Although the author repeatedly emphasized the application of the land footprint to address the environmental impacts of agricultural trade throughout the paper, it lacks quantitative research and qualitative analysis. | The primary aim of this study is to assess the UK’s global land footprint and explore how this could change in the future under difference scenario assumptions. While we discuss and emphasise the importance of estimating land footprints to measure the environmental impacts of consumption, estimating these impacts (for example carbon emissions and biodiversity) is beyond the scope of this study. Future work could explore this aspect using these results. While our aim was not to explore the environmental impacts, we believe it was important to discuss the relevance of our work within the broader environmental impact literature. |

## References

Alexander, P., Arneth, A., Henry, R., Maire, J., Rabin, S., & Rounsevell, M. D. A. (2023). High energy and fertilizer prices are more damaging than food export curtailment from Ukraine and Russia for food prices, health and the environment. *Nature Food*, *4*(1), Article 1. https://doi.org/10.1038/s43016-022-00659-9

Alexander, P., Rabin, S., Anthoni, P., Henry, R., Pugh, T. A. M., Rounsevell, M. D. A., & Arneth, A. (2018). Adaptation of global land use and management intensity to changes in climate and atmospheric carbon dioxide. *Global Change Biology*, *24*(7), 2791–2809. https://doi.org/10.1111/gcb.14110

Gouel, C., & Guimbard, H. (2019). Nutrition Transition and the Structure of Global Food Demand. *American Journal of Agricultural Economics*, *101*(2), 383–403. https://doi.org/10.1093/ajae/aay030

Ma, J., Olin, S., Anthoni, P., Rabin, S. S., Bayer, A. D., Nyawira, S. S., & Arneth, A. (2022). Modeling symbiotic biological nitrogen fixation in grain legumes globally with LPJ-GUESS (v4.0, r10285). *Geoscientific Model Development*, *15*(2), 815–839. https://doi.org/10.5194/gmd-15-815-2022

Müller, C., Franke, J., Jägermeyr, J., Ruane, A. C., Elliott, J., Moyer, E., Heinke, J., Falloon, P. D., Folberth, C., Francois, L., Hank, T., Izaurralde, R. C., Jacquemin, I., Liu, W., Olin, S., Pugh, T. A. M., Williams, K., & Zabel, F. (2021). Exploring uncertainties in global crop yield projections in a large ensemble of crop models and CMIP5 and CMIP6 climate scenarios. *Environmental Research Letters*, *16*(3), 034040. https://doi.org/10.1088/1748-9326/abd8fc

Olin, S., Schurgers, G., Lindeskog, M., Wårlind, D., Smith, B., Bodin, P., Holmér, J., & Arneth, A. (2015). Modelling the response of yields and tissue C: N to changes in atmospheric CO_2_ and N management in the main wheat regions of western Europe. *Biogeosciences*, *12*(8), Article 8. https://doi.org/10.5194/bg-12-2489-2015
