# Supplementary material for: Global land footprint of UK food and feed imports under future socioeconomic scenarios
Source: PLoS One. 2026 Jun 30;21(6):e0352499. doi: 10.1371/journal.pone.0352499 (PMC13318043; doi:10.1371/journal.pone.0352499)
Supplement: Attachment — Submitted filename: Response_to_Reviewers_auresp_2.docx [file pone.0352499.s004.docx]

## Response to reviewers for manuscript “Global land footprint of UK food and feed imports under future socioeconomic scenarios”

We thank the reviewers for their valuable time and feedback on this manuscript. Please find below our responses to all current comments.

*1. The authors have supplemented the paper with relevant literature to explain the model’s national-scale demand-side parameters, the validation basis for LPJ-GUESS, and the spatial resolution of LandSyMM. However, we recommend that the authors further distinguish between two levels in the main text. First, the structural validity of the model at the global scale. Second, the applicable scope when using it for analysis of a single country such as the UK. In particular, the authors should state more explicitly that the paper’s conclusions are better suited for identifying overall trends and relative changes rather than for high-precision national-level forecasts.*

- We agree that structural uncertainty is a fundamental limitation of analyses involving specific mathematical models. Inter-model comparison projects (e.g. AgMIP) are therefore essential to better assess structural uncertainty, and this is an important focus for future research. LandSyMM is the first modelling framework to specifically explore the UK’s land footprint futures and as such we are unable to make comparisons with other models at present. However, the literature presents a wealth of general global land use modelling results from a diversity of modelling frameworks which have been compared in (Alexander et al., 2017; Moallemi et al., 2025). Such approaches, particularly Integrated Assessment Models (IAMs) are prominent in key global environmental assessments including the IPCC’s reports, and have a long history in policy planning. Our work builds on this research, focusing on a UK-specific case study to illustrate the potential for global land use modelling to explore future environmental impacts of food consumption. Our study should not be seen as the final definitive assessment of the UK’s land footprint but rather a first attempt at exploring this topic. We have revised the manuscript to further clarify that the work should be interpreted as a scenario-based assessment of broad trends and relative differences between futures, rather than a detailed forecast.
- While we understand the reviewer’s concern about single-country analysis through the lens of a global model, we consider the use of a global model necessary for addressing the research questions explored here. LandSyMM captures global trade dynamics, aggregate land use change and cross-regional feedbacks between food demand, agricultural production and commodity markets. The UK’s import footprint is inherently determined by global dynamics and therefore cannot be decoupled from the rest of the world. While a model developed and calibrated specifically for the UK might produce more accurate land use dynamics in the UK, it would not be designed to capture the global interdependencies between food demand and production that are critical to this study. However, we fully agree that our results primarily identify key trends and relative changes rather than implying a high degree of precision.
- We have added additional text to address this comment on lines 170-184 and 472-473.

*2. The authors emphasize that this study is not a predictive assessment, noting that the model results are broadly consistent with historical estimates in terms of aggregate quantities. However, this response does not fully address the concerns regarding the reliability of long-term extrapolation and the lack of sufficient support for uncertainty. Non-predictive research cannot substitute for a substantive explanation of the credible boundaries of long-term scenario simulations. Although the authors mention that the simulation results are “relatively consistent” with historical estimates, the lack of clearer comparison metrics, error characterizations, or validation results is insufficient to support a robust assessment of the conclusions regarding the 2100 time horizon.*

- We agree that describing the study as non-predictive does not, by itself, sufficiently define the credible interpretation of long-term scenario outputs. In addition to our response to the previous comment, we have included additional comparisons between modelled and historical results in the supplementary information file (Figures S11 to S14). We show that our model can reproduce the observed global distribution of agricultural production to a level that is acceptable for this analysis. In particular, we highlight the comparison in Fig S13, which shows a high degree of correlation between the UK’s import footprint estimated from FAO data and that simulated by LandSyMM, with Pearson correlation coefficients of 0.70 for cropland, 0.75 for feed crops, 0.68 for food crops, and 0.72 for pasture. A spatial visualisation of this is also shown in Fig S14. As already mentioned in the manuscript, the model is not constrained to reproduce baseline data exactly. Instead, land use patterns emerge endogenously through modelling of land use decision making, in this case least-cost optimisation. This process-based approach allows the model to respond dynamically to changes in demand, trade balances and environmental conditions (e.g. yields). Parts of this comment have been incorporated into the manuscript (lines 178-184). Additional clarifications have been added on lines 462-473.

## References

Alexander, P., Prestele, R., Verburg, P. H., Arneth, A., Baranzelli, C., Silva, F. B. e, Brown, C., Butler, A., Calvin, K., Dendoncker, N., Doelman, J. C., Dunford, R., Engström, K., Eitelberg, D., Fujimori, S., Harrison, P. A., Hasegawa, T., Havlik, P., Holzhauer, S., … Rounsevell, M. D. A. (2017). Assessing uncertainties in land cover projections. *Global Change Biology*, *23*(2), 767–781. https://doi.org/10.1111/gcb.13447

Moallemi, E. A., Castonguay, A. C., Mason-D’Croz, D., Nelson, R., Britz, W., Allen, C., Hadjikakou, M., Battaglia, M., Bryan, B. A., Conti, C., Marcos-Martinez, R., Frank, S., Nong, D., Eker, S., Razavi, S., Navarro-Garcia, J., & Gao, L. (2025). Complexity and uncertainty in future food system transformation modelling. *Nature Food*, *6*(11), 1008–1019. https://doi.org/10.1038/s43016-025-01257-1
