# Supplementary material for: Global land footprint of UK food and feed imports under future socioeconomic scenarios
Source: PLoS One. 2026 Jun 30;21(6):e0352499. doi: 10.1371/journal.pone.0352499 (PMC13318043; doi:10.1371/journal.pone.0352499)
Supplement: Peer review history

# Peer review: Decision Letter 0

**DOI:** 10.1371/journal.pone.0352499.r001

**Authors:** Zhaoxia Guo

6 Nov 2025

PONE-D-25-11636

Global land footprint of UK food and feed imports under future socioeconomic scenarios

PLOS ONE

Dear Dr. Arendarczyk,

Thank you for submitting your manuscript to PLOS ONE. After careful consideration, we feel that it has merit but does not fully meet PLOS ONE’s publication criteria as it currently stands. Therefore, we invite you to submit a revised version of the manuscript that addresses the points raised during the review process.

Please submit your revised manuscript by Dec 21 2025 11:59PM. If you will need more time than this to complete your revisions, please reply to this message or contact the journal office at plosone@plos.org. When you’re ready to submit your revision, log on to https://www.editorialmanager.com/pone/ and select the 'Submissions Needing Revision' folder to locate your manuscript file.

Please include the following items when submitting your revised manuscript:

- A rebuttal letter that responds to each point raised by the academic editor and reviewer(s). You should upload this letter as a separate file labeled 'Response to Reviewers'.
- A marked-up copy of your manuscript that highlights changes made to the original version. You should upload this as a separate file labeled 'Revised Manuscript with Track Changes'.
- An unmarked version of your revised paper without tracked changes. You should upload this as a separate file labeled 'Manuscript'.

If you would like to make changes to your financial disclosure, please include your updated statement in your cover letter. Guidelines for resubmitting your figure files are available below the reviewer comments at the end of this letter.

If applicable, we recommend that you deposit your laboratory protocols in protocols.io to enhance the reproducibility of your results. Protocols.io assigns your protocol its own identifier (DOI) so that it can be cited independently in the future. For instructions see: https://journals.plos.org/plosone/s/submission-guidelines#loc-laboratory-protocols. Additionally, PLOS ONE offers an option for publishing peer-reviewed Lab Protocol articles, which describe protocols hosted on protocols.io. Read more information on sharing protocols at https://plos.org/protocols?utm_medium=editorial-email&utm_source=authorletters&utm_campaign=protocols.

We look forward to receiving your revised manuscript.

Kind regards,

Zhaoxia Guo

Academic Editor

PLOS ONE

Journal Requirements:

When submitting your revision, we need you to address these additional requirements.

1. Please ensure that your manuscript meets PLOS ONE's style requirements, including those for file naming. The PLOS ONE style templates can be found at

https://journals.plos.org/plosone/s/file?id=wjVg/PLOSOne_formatting_sample_main_body.pdf and

https://journals.plos.org/plosone/s/file?id=ba62/PLOSOne_formatting_sample_title_authors_affiliations.pdf

2. Thank you for stating the following financial disclosure:

[B.A. was funded by the Natural Environment Research Council (NERC) through an E4 DTP studentship (NE/S007407/1) (https://www.ukri.org/councils/nerc).].

Please state what role the funders took in the study.  If the funders had no role, please state: "The funders had no role in study design, data collection and analysis, decision to publish, or preparation of the manuscript."

If this statement is not correct you must amend it as needed.

Please include this amended Role of Funder statement in your cover letter; we will change the online submission form on your behalf.

3. Thank you for uploading your study's underlying data set. Unfortunately, the repository you have noted in your Data Availability statement does not qualify as an acceptable data repository according to PLOS's standards.

At this time, please upload the minimal data set necessary to replicate your study's findings to a stable, public repository (such as figshare or Dryad) and provide us with the relevant URLs, DOIs, or accession numbers that may be used to access these data. For a list of recommended repositories and additional information on PLOS standards for data deposition, please see https://journals.plos.org/plosone/s/recommended-repositories.

4. In the online submission form, you indicated that [The full results and analysis scripts are available at https://doi.org/10.5281/zenodo.10631549. Model input data can be obtained from public sources, including the FAO (https://www.fao.org/faostat), World Bank (https://data.worldbank.org), and IIASA (https://tntcat.iiasa.ac.at/SspDb). The crop and pasture yield potential data from LPJ-GUESS are available on request. The model source code is available at https://git.ecdf.ed.ac.uk/lul/plumv2/-/tree/UKLandFootprintPaper.].

All PLOS journals now require all data underlying the findings described in their manuscript to be freely available to other researchers, either 1. In a public repository, 2. Within the manuscript itself, or 3. Uploaded as supplementary information.

This policy applies to all data except where public deposition would breach compliance with the protocol approved by your research ethics board. If your data cannot be made publicly available for ethical or legal reasons (e.g., public availability would compromise patient privacy), please explain your reasons on resubmission and your exemption request will be escalated for approval.

5. Please include a separate caption for each figure in your manuscript.

6. We note that Figures 4 and 6 in your submission contain [map/satellite] images which may be copyrighted. All PLOS content is published under the Creative Commons Attribution License (CC BY 4.0), which means that the manuscript, images, and Supporting Information files will be freely available online, and any third party is permitted to access, download, copy, distribute, and use these materials in any way, even commercially, with proper attribution. For these reasons, we cannot publish previously copyrighted maps or satellite images created using proprietary data, such as Google software (Google Maps, Street View, and Earth). For more information, see our copyright guidelines: http://journals.plos.org/plosone/s/licenses-and-copyright.

We require you to either (1) present written permission from the copyright holder to publish these figures specifically under the CC BY 4.0 license, or (2) remove the figures from your submission:

1. You may seek permission from the original copyright holder of Figure(s) [#] to publish the content specifically under the CC BY 4.0 license.

We recommend that you contact the original copyright holder with the Content Permission Form (http://journals.plos.org/plosone/s/file?id=7c09/content-permission-form.pdf) and the following text:

“I request permission for the open-access journal PLOS ONE to publish XXX under the Creative Commons Attribution License (CCAL) CC BY 4.0 (http://creativecommons.org/licenses/by/4.0/). Please be aware that this license allows unrestricted use and distribution, even commercially, by third parties. Please reply and provide explicit written permission to publish XXX under a CC BY license and complete the attached form.”

Please upload the completed Content Permission Form or other proof of granted permissions as an "Other" file with your submission.

In the figure caption of the copyrighted figure, please include the following text: “Reprinted from [ref] under a CC BY license, with permission from [name of publisher], original copyright [original copyright year].”

2. If you are unable to obtain permission from the original copyright holder to publish these figures under the CC BY 4.0 license or if the copyright holder’s requirements are incompatible with the CC BY 4.0 license, please either i) remove the figure or ii) supply a replacement figure that complies with the CC BY 4.0 license. Please check copyright information on all replacement figures and update the figure caption with source information. If applicable, please specify in the figure caption text when a figure is similar but not identical to the original image and is therefore for illustrative purposes only.

The following resources for replacing copyrighted map figures may be helpful:

USGS National Map Viewer (public domain): http://viewer.nationalmap.gov/viewer/

The Gateway to Astronaut Photography of Earth (public domain): http://eol.jsc.nasa.gov/sseop/clickmap/

Maps at the CIA (public domain): https://www.cia.gov/library/publications/the-world-factbook/index.html and https://www.cia.gov/library/publications/cia-maps-publications/index.html

NASA Earth Observatory (public domain): http://earthobservatory.nasa.gov/

Landsat: http://landsat.visibleearth.nasa.gov/

USGS EROS (Earth Resources Observatory and Science (EROS) Center) (public domain): http://eros.usgs.gov/#

Natural Earth (public domain): http://www.naturalearthdata.com/

7. If the reviewer comments include a recommendation to cite specific previously published works, please review and evaluate these publications to determine whether they are relevant and should be cited. There is no requirement to cite these works unless the editor has indicated otherwise.

Additional Editor Comments (if provided):

[Note: HTML markup is below. Please do not edit.]

Reviewers' comments:

Reviewer's Responses to Questions

**Comments to the Author**

1. Is the manuscript technically sound, and do the data support the conclusions?

The manuscript must describe a technically sound piece of scientific research with data that supports the conclusions. Experiments must have been conducted rigorously, with appropriate controls, replication, and sample sizes. The conclusions must be drawn appropriately based on the data presented.

Reviewer #1: Yes

Reviewer #2: Partly

**********

2. Has the statistical analysis been performed appropriately and rigorously?

Reviewer #1: Yes

Reviewer #2: Yes

**********

3. Have the authors made all data underlying the findings in their manuscript fully available?

The PLOS Data policy requires authors to make all data underlying the findings described in their manuscript fully available without restriction, with rare exception (please refer to the Data Availability Statement in the manuscript PDF file). The data should be provided as part of the manuscript or its supporting information, or deposited to a public repository. For example, in addition to summary statistics, the data points behind means, medians and variance measures should be available. If there are restrictions on publicly sharing data—e.g. participant privacy or use of data from a third party—those must be specified.

Reviewer #1: Yes

Reviewer #2: Yes

**********

4. Is the manuscript presented in an intelligible fashion and written in standard English?

PLOS ONE does not copyedit accepted manuscripts, so the language in submitted articles must be clear, correct, and unambiguous. Any typographical or grammatical errors should be corrected at revision, so please note any specific errors here.

Reviewer #1: Yes

Reviewer #2: Yes

**********

5. Review Comments to the Author

Please use the space provided to explain your answers to the questions above. You may also include additional comments for the author, including concerns about dual publication, research ethics, or publication ethics. (Please upload your review as an attachment if it exceeds 20,000 characters)

Reviewer #1: This manuscript presents an in-depth analysis of the global land footprint associated with the UK’s food and feed imports, both historically (2010–2020) and under future scenarios up to 2100. Using a combination of historical FAOSTAT data and simulations from the Land System Modular Model (LandSyMM), the authors estimate the land use impacts of UK consumption across four socioeconomic scenarios. The integration of advanced land use modeling with scenario analysis provides valuable insights for both academic researchers and policymakers. However, there are still some contents that need to be carefully revised before final publication.

1. The methodological section is comprehensive but would benefit from greater clarity and conciseness. While the integration of the LandSyMM framework, including LPJ-GUESS and PLUM, is described in detail, summarizing the overall workflow with a schematic or flowchart would make it more accessible and easier to follow.

2. The manuscript employs four SSP-RCP scenarios (SSP1-RCP2.6, SSP3-RCP7.0, SSP4-RCP6.0, and SSP5-RCP8.5) to explore the future global land footprint of UK food and feed imports. However, SSP2, which is widely used in multi-scenario analyses as a reference or “middle-of-the-road” scenario, is notably absent. Why was SSP2 excluded from the analysis, given its common use in scenario-based studies for comparative analysis? The manuscript should provide a clear rationale for excluding SSP2 and explain why the selected scenarios sufficiently capture the spectrum of possible future trajectories.

3. The manuscript assumes that trade preferences remain constant throughout the 21st century, meaning the share of commodities imported from specific exporting countries does not change over time. While this simplifies the modeling process, it is a significant limitation that may not accurately reflect the dynamic nature of international trade influenced by geopolitical, economic, and environmental factors. For example, recent global events, such as the trade wars initiated by the Trump administration (e.g., increasing tariffs on key imports), demonstrate how trade preferences and patterns can shift rapidly due to policy decisions. Similarly, the ongoing emphasis on reducing reliance on specific regions for critical goods (e.g., reducing dependency on China for supply chains) or addressing environmental concerns (e.g., the EU’s deforestation-free trade regulations) suggests that trade preferences are likely to evolve significantly in the future. The manuscript should explicitly discuss the limitations of assuming constant trade preferences and how this assumption might affect the reliability of the results. For instance, how would shifts in trade relationships (e.g., increased tariffs, sanctions, or new trade agreements) impact the projected land footprint?

4. Figure 1 shows that in SSP1 and SSP4, which represent shifts towards healthier and more sustainable diets (e.g., increased consumption of fruits and vegetables and reduced consumption of animal products), the projected imports of vegetables and fruits from 2080 to 2100 are smaller than those in SSP3, which assumes no dietary shift. This trend is counterintuitive, as healthier diets are expected to increase fruit and vegetable consumption. Could this result be explained by differences in population growth across the scenarios? For example, SSP3 assumes higher population growth, which might offset the lower demand per capita. However, the manuscript does not explicitly present the population trajectories or other key socioeconomic drivers for each scenario, making it difficult to interpret the underlying causes of this trend. In addition, the results for C4 cereals, other oil crops, N-fixing oil crops, rice, and pulses show remarkably small differences across the four scenarios. This contrasts with the significant variation observed for other food groups, such as fruits, vegetables, and animal products. The manuscript does not explain why these crop categories are relatively unaffected by the dietary shifts and socioeconomic changes modeled in the scenarios. Is this due to inherent assumptions in the LandSyMM model (e.g., limited scope for change in demand or production for these crops), or are these crops less sensitive to population and dietary shifts in the UK?

5. The manuscript provides detailed projections of the UK’s land footprint under different SSP-RCP scenarios, but some results lack clear and convincing explanations, which raises concerns about the robustness and interpretability of the findings. For instance, regarding lines 452–458, the results indicate that despite higher population growth and increased demand for animal products in SSP3, the land footprint does not increase as one might intuitively expect. Instead, the SSP3 land footprint remains comparable to or even slightly lower than other scenarios. Although some justification is offered in lines 460–464—namely, that higher agricultural intensity mitigates land footprint growth—this explanation is not fully convincing or substantiated within the current manuscript. Given the centrality of this counterintuitive result, I believe the authors should provide a much more thorough and transparent analysis. Specifically, the manuscript should: 1) clearly describe the mechanisms in the model that allow increased demand to be offset by agricultural intensification; 2) present supporting data or sensitivity analyses demonstrating how changes in yields, management practices, or trade patterns in SSP3 lead to a stable or reduced land footprint; 3) compare these findings more directly to previous literature and discuss to what extent they are consistent or divergent.

Reviewer #2: This manuscript, entitled ‘Global land footprint of UK food and feed imports under future socioeconomic scenario’ (PONE-D-25-11636), represents the first systematic assessment of the global land footprint of the UK's food and feed imports, combining historical data with future scenario simulations. It demonstrates clear practical significance and methodological innovation. The research design is well-structured, employing advanced methodologies with transparent data sources. There are some obvious problems, and I would suggest that they be significantly revised before further consideration.

1. It is unclear whether the global-scale land system model LandSyMM possesses sufficient resolution and accuracy at the national level (e.g., the United Kingdom). It is recommended to supplement the model with validation of its applicability at the national scale or supporting literature.

2. It is necessary to supplement the technology roadmap and detailed explanations. This helps readers from other fields quickly grasp the research structure and methodology.

3. It is recommended to insert spatial distribution maps of land footprints for key years or scenarios into the main text to enhance visual representation and policy interpretation capabilities.

4. The model employs historical data from 2010 to 2020 and comprehensive scenario assumptions to project long-term land footprint trends from 2020 to 2100. However, a significant concern arises: can the limited historical data and numerous assumptions sufficiently support the reliability of such extended-term projections? The authors must ensure enhanced the article's findings.

5. Although “stochastically sampled input parameters” are mentioned, the study does not specify which parameters were sampled, the sampling distribution used, or whether sensitivity analysis was performed. It is recommended to supplement the details of the uncertainty analysis to enhance the credibility of the results.

6. The author conducted historical research and projections on the food land footprint generated by British feed imports, but did not delve deeply into the resource and environmental impacts resulting from trade imports. Although the author repeatedly emphasized the application of the land footprint to address the environmental impacts of agricultural trade throughout the paper, it lacks quantitative research and qualitative analysis.

**********

6. PLOS authors have the option to publish the peer review history of their article (what does this mean?). If published, this will include your full peer review and any attached files.

If you choose “no”, your identity will remain anonymous but your review may still be made public.

**Do you want your identity to be public for this peer review?**  For information about this choice, including consent withdrawal, please see our Privacy Policy.

Reviewer #1: No

Reviewer #2: No

**********

[NOTE: If reviewer comments were submitted as an attachment file, they will be attached to this email and accessible via the submission site. Please log into your account, locate the manuscript record, and check for the action link "View Attachments". If this link does not appear, there are no attachment files.]

To ensure your figures meet our technical requirements, please review our figure guidelines: https://journals.plos.org/plosone/s/figures

You may also use PLOS’s free figure tool, NAAS, to help you prepare publication quality figures: https://journals.plos.org/plosone/s/figures#loc-tools-for-figure-preparation.

NAAS will assess whether your figures meet our technical requirements by comparing each figure against our figure specifications.

# Author comment: Author response to Decision Letter 1

**DOI:** 10.1371/journal.pone.0352499.r002

20 Jan 2026

Response to reviewers is included in file 'Response to Reviewers.docx'

Attachment Submitted filename: Response To Reviewers.docx

# Peer review: Decision Letter 1

**DOI:** 10.1371/journal.pone.0352499.r003

**Authors:** Zhaoxia Guo, Zhaoxia Guo

24 Apr 2026

PONE-D-25-11636R1

Global land footprint of UK food and feed imports under future socioeconomic scenarios

PLOS One

Dear Dr. Arendarczyk,

Thank you for submitting your manuscript to PLOS ONE. After careful consideration, we feel that it has merit but does not fully meet PLOS ONE’s publication criteria as it currently stands. Therefore, we invite you to submit a revised version of the manuscript that addresses the points raised during the review process.

Please submit your revised manuscript by Jun 07 2026 11:59PM. If you will need more time than this to complete your revisions, please reply to this message or contact the journal office at plosone@plos.org. When you're ready to submit your revision, log on to https://www.editorialmanager.com/pone/ and select the 'Submissions Needing Revision' folder to locate your manuscript file.

Please include the following items when submitting your revised manuscript:

- A letter that responds to each point raised by the academic editor and reviewer(s). You should upload this letter as a separate file labeled 'Response to Reviewers'.
- A marked-up copy of your manuscript that highlights changes made to the original version. You should upload this as a separate file labeled 'Revised Manuscript with Track Changes'.
- An unmarked version of your revised paper without tracked changes. You should upload this as a separate file labeled 'Manuscript'.

If you would like to make changes to your financial disclosure, please include your updated statement in your cover letter. Guidelines for resubmitting your figure files are available below the reviewer comments at the end of this letter.

If applicable, we recommend that you deposit your laboratory protocols in protocols.io to enhance the reproducibility of your results. Protocols.io assigns your protocol its own identifier (DOI) so that it can be cited independently in the future. For instructions see: https://journals.plos.org/plosone/s/submission-guidelines#loc-laboratory-protocols. Additionally, PLOS ONE offers an option for publishing peer-reviewed Lab Protocol articles, which describe protocols hosted on protocols.io. Read more information on sharing protocols at https://plos.org/protocols?utm_medium=editorial-email&utm_source=authorletters&utm_campaign=protocols.

As the corresponding author, your ORCID iD is verified in the submission system and will appear in the published article. PLOS supports the use of ORCID, and we encourage all coauthors to register for an ORCID iD and use it as well. Please encourage your coauthors to verify their ORCID iD within the submission system before final acceptance, as unverified ORCID iDs will not appear in the published article. *Only*  the individual author can complete the verification step; PLOS staff *cannot*  verify ORCID iDs on behalf of authors.

We look forward to receiving your revised manuscript.

Kind regards,

Zhaoxia Guo

Academic Editor

PLOS One

Journal Requirements:

1. If the reviewer comments include a recommendation to cite specific previously published works, please review and evaluate these publications to determine whether they are relevant and should be cited. There is no requirement to cite these works unless the editor has indicated otherwise.

2. Please review your reference list to ensure that it is complete and correct. If you have cited papers that have been retracted, please include the rationale for doing so in the manuscript text, or remove these references and replace them with relevant current references. Any changes to the reference list should be mentioned in the rebuttal letter that accompanies your revised manuscript. If you need to cite a retracted article, indicate the article’s retracted status in the References list and also include a citation and full reference for the retraction notice.

[Note: HTML markup is below. Please do not edit.]

Reviewers' comments:

Reviewer's Responses to Questions

**Comments to the Author**

1. If the authors have adequately addressed your comments raised in a previous round of review and you feel that this manuscript is now acceptable for publication, you may indicate that here to bypass the “Comments to the Author” section, enter your conflict of interest statement in the “Confidential to Editor” section, and submit your "Accept" recommendation.

Reviewer #1: All comments have been addressed

Reviewer #2: (No Response)

**********

2. Is the manuscript technically sound, and do the data support the conclusions?

The manuscript must describe a technically sound piece of scientific research with data that supports the conclusions. Experiments must have been conducted rigorously, with appropriate controls, replication, and sample sizes. The conclusions must be drawn appropriately based on the data presented.

Reviewer #1: Yes

Reviewer #2: Partly

**********

3. Has the statistical analysis been performed appropriately and rigorously?

Reviewer #1: Yes

Reviewer #2: Yes

**********

4. Have the authors made all data underlying the findings in their manuscript fully available?

The PLOS Data policy requires authors to make all data underlying the findings described in their manuscript fully available without restriction, with rare exception (please refer to the Data Availability Statement in the manuscript PDF file). The data should be provided as part of the manuscript or its supporting information, or deposited to a public repository. For example, in addition to summary statistics, the data points behind means, medians and variance measures should be available. If there are restrictions on publicly sharing data—e.g. participant privacy or use of data from a third party—those must be specified.

Reviewer #1: Yes

Reviewer #2: Yes

**********

5. Is the manuscript presented in an intelligible fashion and written in standard English?

PLOS ONE does not copyedit accepted manuscripts, so the language in submitted articles must be clear, correct, and unambiguous. Any typographical or grammatical errors should be corrected at revision, so please note any specific errors here.

Reviewer #1: Yes

Reviewer #2: Yes

**********

6. Review Comments to the Author

Please use the space provided to explain your answers to the questions above. You may also include additional comments for the author, including concerns about dual publication, research ethics, or publication ethics. (Please upload your review as an attachment if it exceeds 20,000 characters)

Reviewer #1: (No Response)

Reviewer #2: The authors have made significant revisions to the manuscript (PONE-D-25-11636_R1), but some issues remain that require further clarification.

1. The authors have supplemented the paper with relevant literature to explain the model’s national-scale demand-side parameters, the validation basis for LPJ-GUESS, and the spatial resolution of LandSyMM. However, we recommend that the authors further distinguish between two levels in the main text. First, the structural validity of the model at the global scale. Second, the applicable scope when using it for analysis of a single country such as the UK. In particular, the authors should state more explicitly that the paper’s conclusions are better suited for identifying overall trends and relative changes rather than for high-precision national-level forecasts.

2. The authors emphasize that this study is not a predictive assessment, noting that the model results are broadly consistent with historical estimates in terms of aggregate quantities. However, this response does not fully address the concerns regarding the reliability of long-term extrapolation and the lack of sufficient support for uncertainty. Non-predictive research cannot substitute for a substantive explanation of the credible boundaries of long-term scenario simulations. Although the authors mention that the simulation results are “relatively consistent” with historical estimates, the lack of clearer comparison metrics, error characterizations, or validation results is insufficient to support a robust assessment of the conclusions regarding the 2100 time horizon.

**********

7. PLOS authors have the option to publish the peer review history of their article (what does this mean?). If published, this will include your full peer review and any attached files.

If you choose “no”, your identity will remain anonymous but your review may still be made public.

**Do you want your identity to be public for this peer review?**  For information about this choice, including consent withdrawal, please see our Privacy Policy.

Reviewer #1: No

Reviewer #2: No

**********

[NOTE: If reviewer comments were submitted as an attachment file, they will be attached to this email and accessible via the submission site. Please log into your account, locate the manuscript record, and check for the action link "View Attachments". If this link does not appear, there are no attachment files.]

To ensure your figures meet our technical requirements, please review our figure guidelines: https://journals.plos.org/plosone/s/figures

You may also use PLOS’s free figure tool, NAAS, to help you prepare publication quality figures: https://journals.plos.org/plosone/s/figures#loc-tools-for-figure-preparation.

NAAS will assess whether your figures meet our technical requirements by comparing each figure against our figure specifications.

# Author comment: Author response to Decision Letter 2

**DOI:** 10.1371/journal.pone.0352499.r004

19 May 2026

Please see the included "Response to Reviewers.docx" document.

Attachment Submitted filename: Response_to_Reviewers_auresp_2.docx

# Peer review: Decision Letter 2

**DOI:** 10.1371/journal.pone.0352499.r005

**Authors:** Zhaoxia Guo, Zhaoxia Guo, Zhaoxia Guo

11 Jun 2026

Global land footprint of UK food and feed imports under future socioeconomic scenarios

PONE-D-25-11636R2

Dear Dr. Arendarczyk,

We’re pleased to inform you that your manuscript has been judged scientifically suitable for publication and will be formally accepted for publication once it meets all outstanding technical requirements.

Within one week, you’ll receive an e-mail detailing the required amendments. When these have been addressed, you’ll receive a formal acceptance letter and your manuscript will be scheduled for publication.

An invoice will be generated when your article is formally accepted. Please note, if your institution has a publishing partnership with PLOS and your article meets the relevant criteria, all or part of your publication costs will be covered. Please make sure your user information is up-to-date by logging into Editorial Manager at Editorial Manager® and clicking the ‘Update My Information' link at the top of the page. For questions related to billing, please contact billing support.

If your institution or institutions have a press office, please notify them about your upcoming paper to help maximize its impact. If they’ll be preparing press materials, please inform our press team as soon as possible -- no later than 48 hours after receiving the formal acceptance. Your manuscript will remain under strict press embargo until 2 pm Eastern Time on the date of publication. For more information, please contact onepress@plos.org.

Kind regards,

Zhaoxia Guo

Academic Editor

PLOS One

# Editor report: Acceptance letter

**DOI:** 10.1371/journal.pone.0352499.r006

**Authors:** Zhaoxia Guo, Zhaoxia Guo, Zhaoxia Guo

PONE-D-25-11636R2

PLOS One

Dear Dr. Arendarczyk,

I'm pleased to inform you that your manuscript has been deemed suitable for publication in PLOS One. Congratulations! Your manuscript is now being handed over to our production team.

At this stage, our production department will prepare your paper for publication. This includes ensuring the following:

* All references, tables, and figures are properly cited

* All relevant supporting information is included in the manuscript submission,

* There are no issues that prevent the paper from being properly typeset

You will receive further instructions from the production team, including instructions on how to review your proof when it is ready. Please keep in mind that we are working through a large volume of accepted articles, so please give us a few days to review your paper and let you know the next and final steps.

Lastly, if your institution or institutions have a press office, please let them know about your upcoming paper now to help maximize its impact. If they'll be preparing press materials, please inform our press team within the next 48 hours. Your manuscript will remain under strict press embargo until 2 pm Eastern Time on the date of publication. For more information, please contact onepress@plos.org.

You will receive an invoice from PLOS for your publication fee after your manuscript has reached the completed accept phase. If you receive an email requesting payment before acceptance or for any other service, this may be a phishing scheme. Learn how to identify phishing emails and protect your accounts at https://explore.plos.org/phishing.

If we can help with anything else, please email us at customercare@plos.org.

Thank you for submitting your work to PLOS ONE and supporting open access.

Kind regards,

PLOS ONE Editorial Office Staff

on behalf of

Dr. Zhaoxia Guo

Academic Editor

PLOS One
